# Archean (3.3 Ga) paleosols and paleoenvironments of Western Australia

**DOI:** 10.1371/journal.pone.0291074

**Published:** 2023-09-27

**Authors:** Gregory J. Retallack, Mark D. Schmitz

**Affiliations:** 1 Department of Earth Sciences, University of Oregon, Eugene, Oregon, United States of America; 2 Department of Geoscience, Boise State University, Boise, Idaho, United States of America; Ben-Gurion University of the Negev, ISRAEL

## Abstract

The Pilbara craton of northwestern Australia is known for what were, when reported, the oldest known microfossils and paleosols on Earth. Both interpretations are mired in controversy, and neither remain the oldest known. Both the microfossils and the paleosols have been considered hydrothermal artefacts: carbon films of vents and a large hydrothermal cupola, respectively. This study resampled and analyzed putative paleosols within and below the Strelley Pool Formation (3.3 Ga), at four classic locations: Strelley Pool, Steer Ridge, Trendall Ridge, and Streckfuss, and also at newly discovered outcrops near Marble Bar. The same sequence of sedimentary facies and paleosols was newly recognized unconformably above the locality for microfossils in chert of the Apex Basalt (3.5 Ga) near Marble Bar. The fossiliferous Apex chert was not a hydrothermal vein but a thick (15 m) sedimentary interbed within a sequence of pillow basalts, which form an angular unconformity capped by the same pre-Strelley paleosol and Strelley Pool Formation facies found elsewhere in the Pilbara region. Baritic alluvial paleosols within the Strelley Pool Formation include common microfossil spindles (cf. *Eopoikilofusa*) distinct from marine microfossil communities with septate filaments (*Primaevifilum*) of cherts in the Apex and Mt Ada Basalts. Phosphorus and iron depletion in paleosols within and below the Strelley Pool Formation are evidence of soil communities of stable landscapes living under an atmosphere of high CO_2_ (2473 ± 134 ppmv or 8.8 ± 0.5 times preindustrial atmospheric level of 280 ppm) and low O_2_ (2181 ± 3018 ppmv or 0.01 ± 0.014 times modern).

## Introduction

The possibility of new evidence for Archean life on land, as well as for the nature of Archean atmosphere and climate, was opened by discovery in 1995 of a thick and distinctive paleosol beneath the 3.3 Ga Strelley Pool Formation of the Pilbara Desert, Western Australia. The pre-Strelley paleosol was thought to extend 50 m below a regional angular unconformity and taken as evidence for a surprisingly large ancient landmass [1]. The hope for more paleoenvironmental and paleobiological information was frustrated when the paleosol was later interpreted as an exhumed hydrothermal cupola some 80 m thick around the North Pole Monzogranite and associated granitic dikes [2, 3]. Subsequently the paleosol was again interpreted as a deep weathering profile, but 35 m thick and including highly oxidized laterite, with implications for a surprisingly oxidizing (>0.48 times present O_2_) Archean atmosphere [4]. This study attempts to reconcile these differences and explore more fully evidence for Archean life and paleoclimate with the first detailed geochemical and petrographic study of the pre-Strelley surface at five localities, including newly recognized exposures directly above the controversial Apex chert microfossil locality near Marble Bar [5, 6].

Discovery of Strelley Pool Formation near Marble Bar reported here, is also relevant to interpretation of marine microfossils from the Apex Chert and Strelley Pool Formation there. Brasier et al. [6–9] attacked previous interpretations by arguing that microfossils of the 3.46 Ga Apex Chert were indeterminate skeins of abiotically produced carbon from a hydrothermal feeder to a submarine vent. Prior publication in contrast, regarded microfossils in cherts near Marble Bar as genuine [10], a position supported by additional studies of Raman spectroscopy, carbon isotopes, three-dimensional imaging [1, 11], and nanostructural data [12, 13]. The Apex chert microfossils are no longer the oldest evidence of life [14–17], but paleoenvironmental context relevant to Apex microfossils is also re-examined here as evidence for differences between Archean microbiomes on land and at sea.

### Geological background

**Fig 1:**
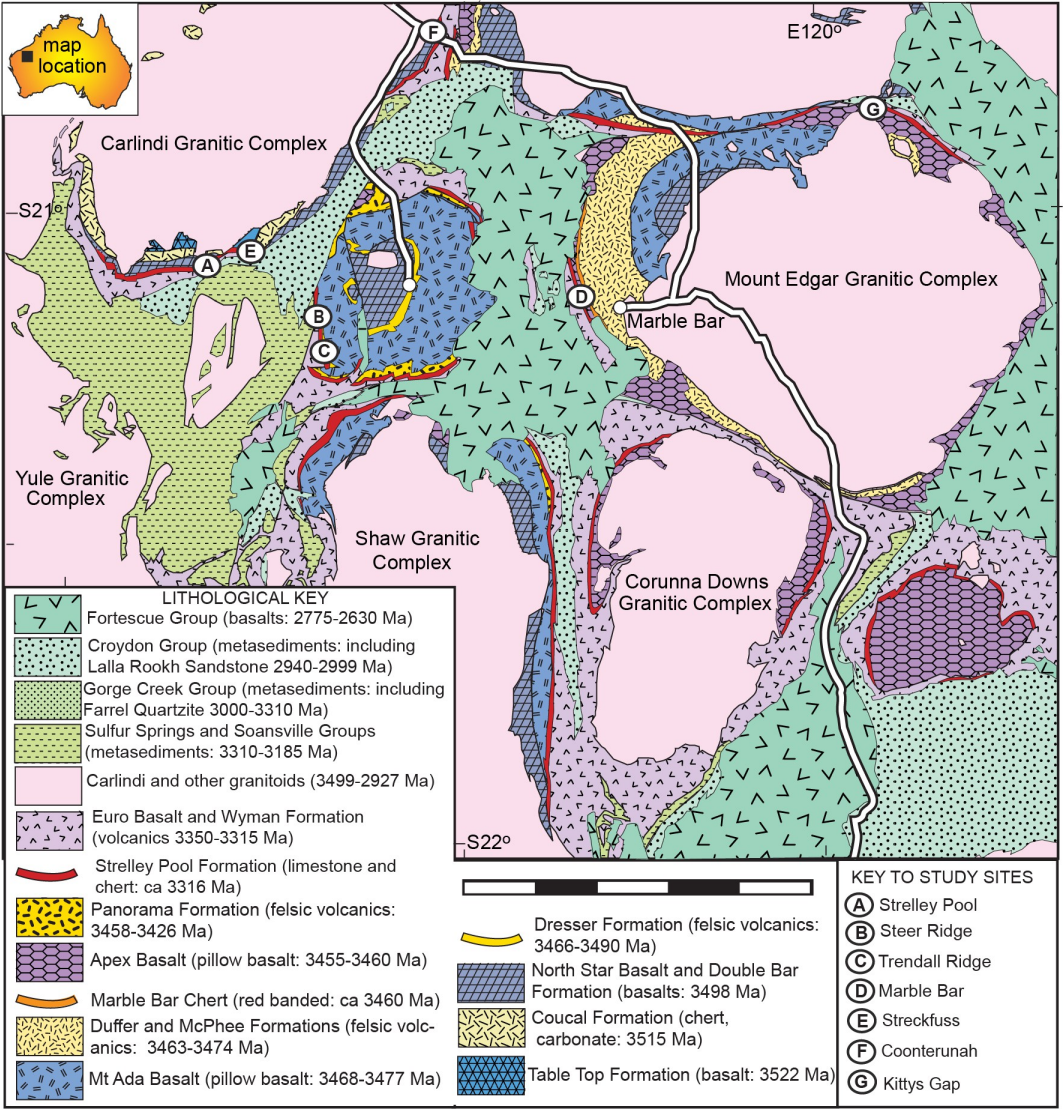
Simplified geological map of the Strelley Pool Formation, among other Archean formations in Western Australia, showing newly proposed distribution combined with previously published mapping [7, 46, 47, 49].

**Fig 2:**
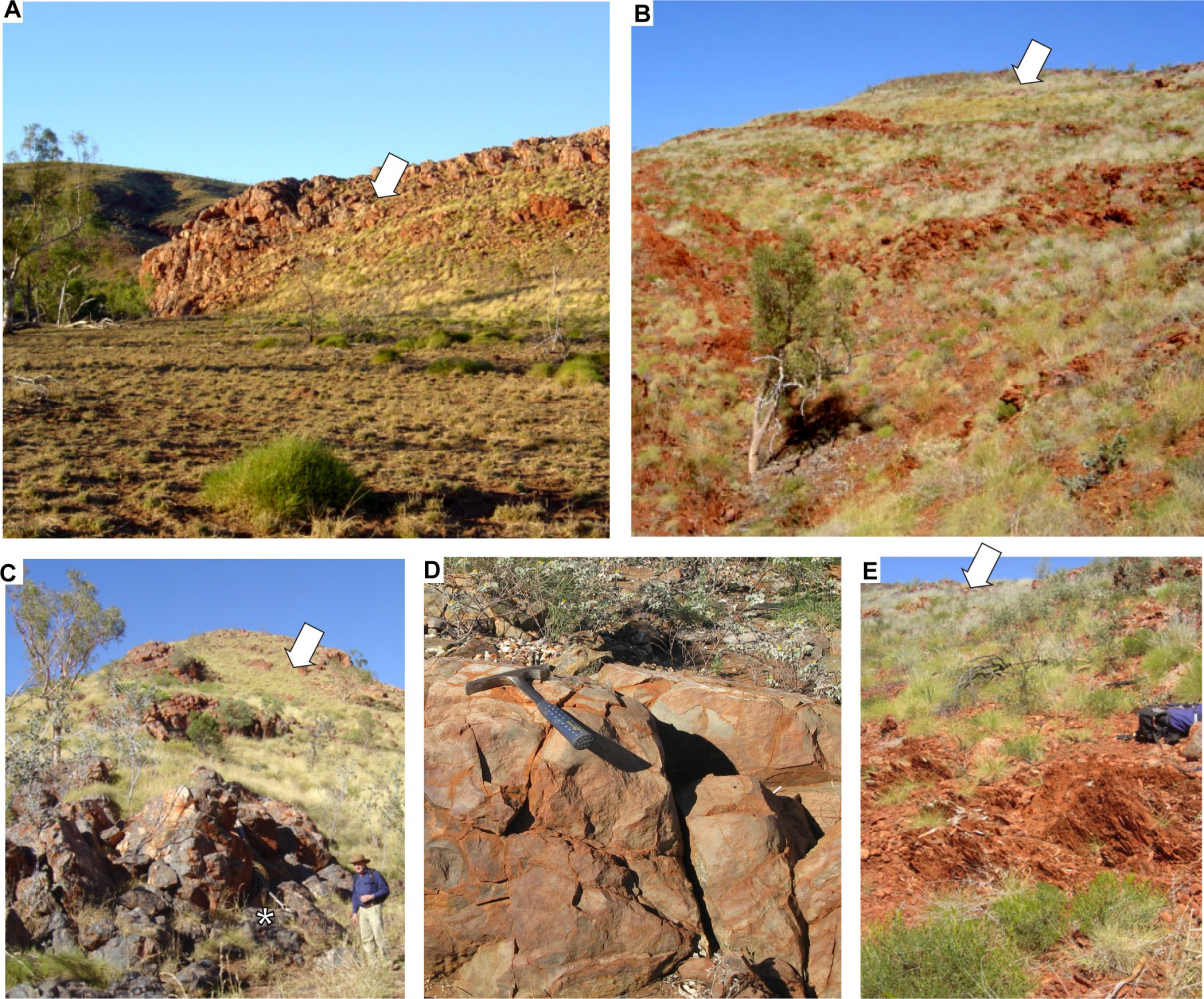
Study locations (arrows) for the pre-Strelley paleosol west of Strelley Pool (**A**), on Trendall Ridge (**B**), and above Schopf microfossil locality in Apex chert west of Marble Bar (**C**), as well as footwall lithologies: pillowed Apex Basalt west of Marble Bar (**D**) and ferruginous tuffaceous siltstone of Panorama Formation at Trendall Ridge (**E**). Asterisk on Apex Chert is the original microfossil site [10] resampled during this work for study of graded bedding, intraclasts and microfossils. Hammers for scale are 25 cm, and backpack is 40 cm wide.

**Fig 3:**
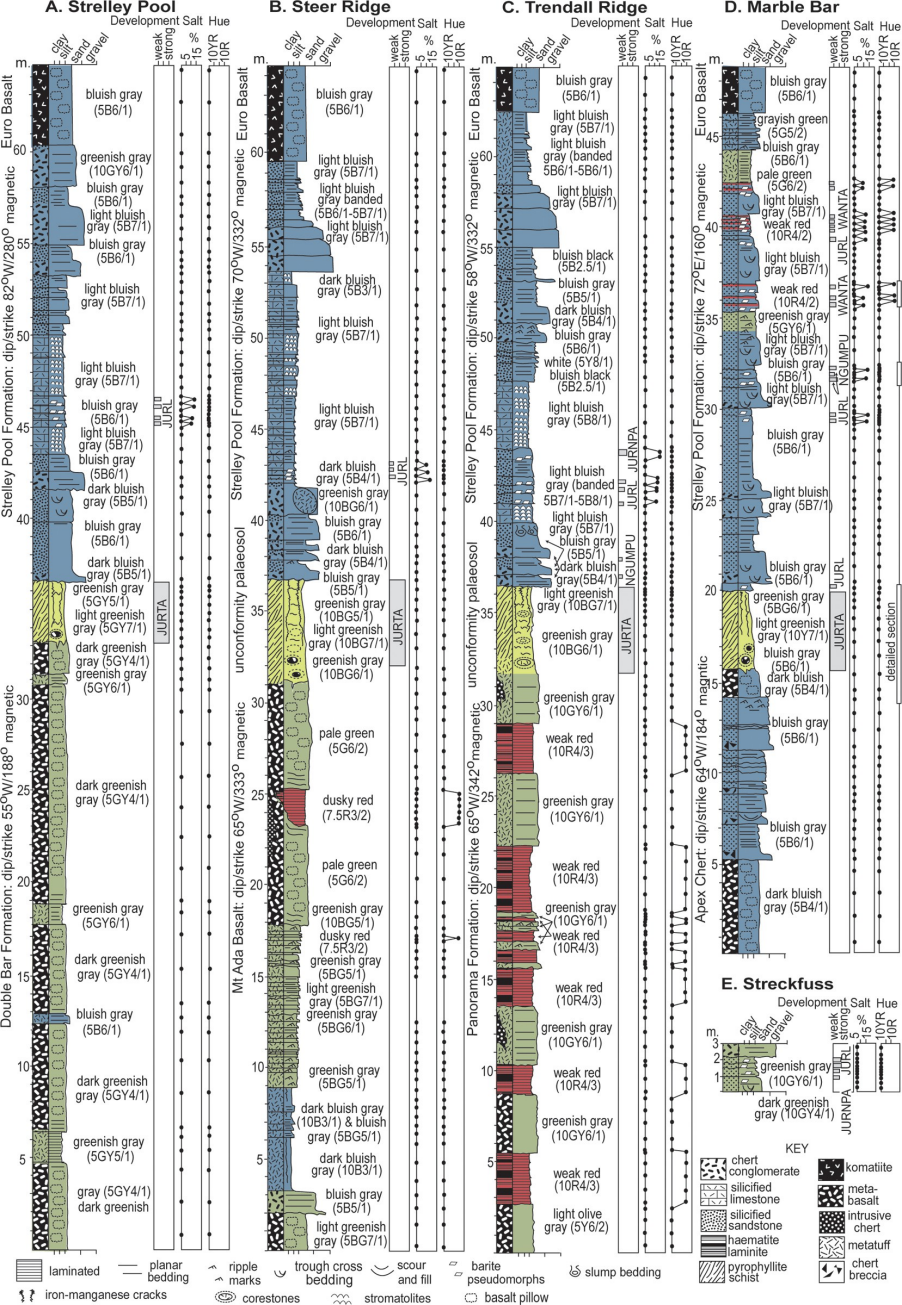
Measured sections of Strelley Pool Formation and the pre-Strelley paleosol.

**Fig 4:**
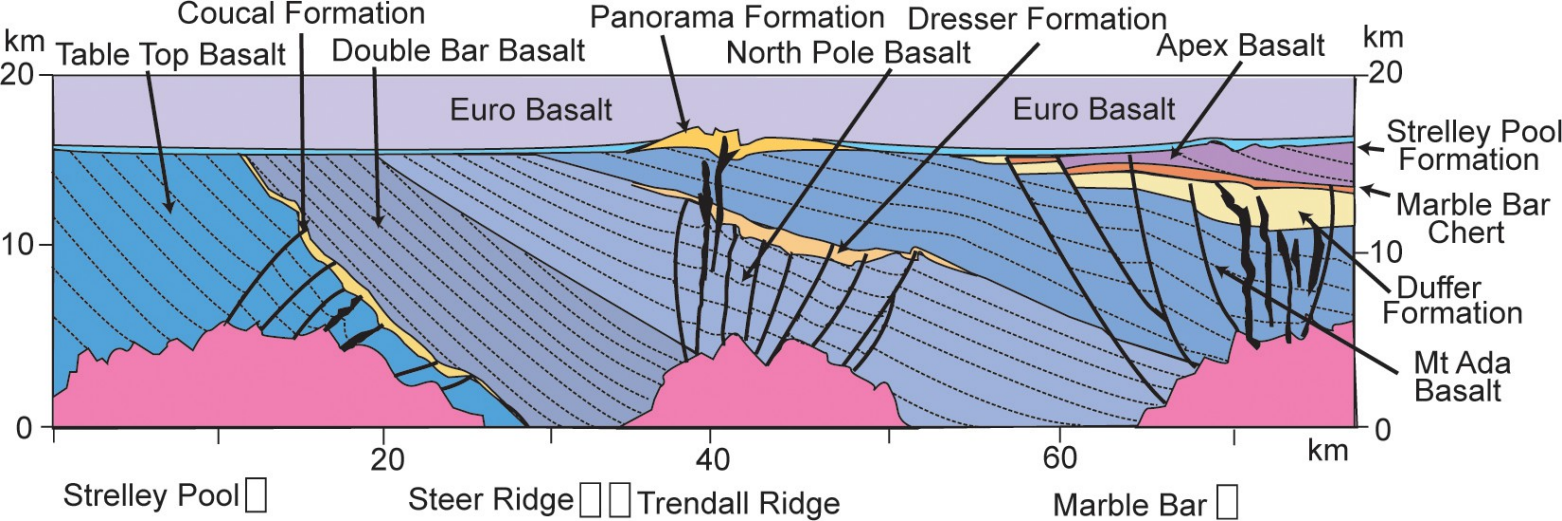
Restoration of geological relationships on an east-west cross section of the east Pilbara craton during eruption of Euro flood basalts at 3420 Ma. The Strelley Pool Formation represents non-volcanic sedimentation following a hiatus of millions of years and formation of the 4 m deep pre-Strelley paleosol.

**Fig 5:**
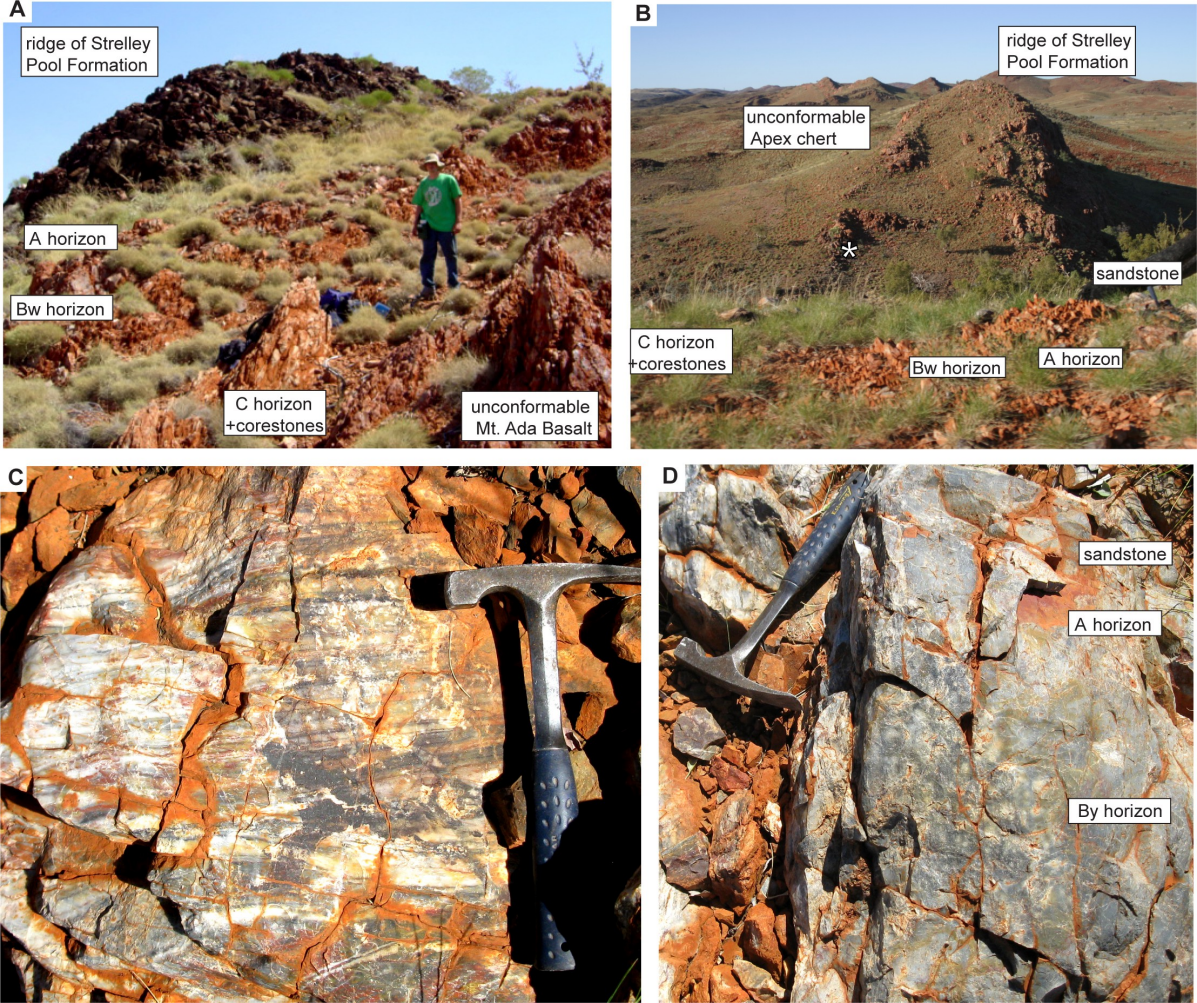
Field photographs of pre-Strelley Jurta paleosol (**A, B**) and sediments (**C**) and Jurl paleosol (**D**) within the Strelley Pool Formation: (**A**) Jurta paleosol on Steer Ridge; (**B**) Jurta paleosol west of Marble Bar pool; (**C**) trough cross bedding at 31 m in Marble Bar section: (**D**) Jurl paleosol with barite sand crystals at 32 m in Marble Bar section. Asterisk in (A) is microfossil locality of Schopf and Packer (1987). Ian Johnson for scale (A), and hammers 25 cm long for scale (C, D).

The Strelley Pool Formation is generally 28 m thick over some 30,000 km^2^ of the East Pilbara Craton of Western Australia (Fig 1). Intrusion of granitic complexes has deformed the Strelley Pool Formation to near vertical so that it crops out as ridges (Fig 2A–2C and 2E). The formation has a similar succession of facies over its entire area (Fig 3A–3D), and is famous for stromatolites [18–20] and a variety of microfossils [8, 9, 21–25]. The angular unconformity beneath the Strelley Pool Formation (Fig 4) has a 4–6 m thick layer of sericite (Fig 5A and 5B), widely interpreted as a paleosol [1, 4]. This study provides the first detailed profile data on that paleosol, but also describes in detail paleosols within the Strelley Pool Formation (Figs 3 and 5D), comparable with other Archean paleosols from Western Australia [26, 27].

A lower limit for geological age of the Strelley Pool Formation comes from dacite of the Kangaroo Caves Formation of the Sulphur Springs Group, 9 km higher in the sequence, dated by U-Pb on zircon as 3240 ± 2 Ma [28]. An upper limit is from rhyolite zircons of the underlying Panorama Formation, dated in Glen Herring Creek by U-Pb at 3430 ± 4 Ma [27], and on Panorama Ridge at 3458 ± 1.9 Ma [28]. Also unconformably beneath the Strelley Pool Formation near Marble Bar is Apex Basalt onlapping Marble Bar Chert above Duffer Formation dated at 3466 ± 2 Ma [29]. Near Steer Ridge the rock below the sub-Strelley unconformity is Mount Ada Basalt dated at 3449 ± 3 and 3522 ±18 Ma [26, 30], and near Strelley Pool it is on Double Bar Basalt, conformably overlying rhyolite of the Coucal Formation dated at 3515 ± 2 Ma [1]. U-Pb age of detrital zircons near Strelley Pool gave maximal deposition ages of the Strelley Pool Formation of 3502 ± 3 Ma and 3479 ± 8 Ma [31], and zircons near the base of the Euro Basalt gave ages of 3350 ± 3 Ma [32–34].

## Materials and methods

### Study sites

This study focuses on paleoenvironmental interpretation of the Strelley Pool Formation and the thick paleosol formed on rocks of its basal angular unconformity at five localities (Fig 1): Strelley Pool (S21.11094^o^ E119.13709^o^), Steer Ridge (S21.18848^o^ E119.30154^o^), Trendall Ridge (S21.18848^o^ E119.30154^o^), Streckfuss (S20.82048^o^ E119.49197^o^), and Marble Bar (S21.17603^o^ E119.69779^o^). Additional information regarding the ethical, cultural, and scientific considerations specific to inclusivity in global research is included in the S1 File. The Strelley Pool section was measured on the north face of the ridge 300 m west of Strelley Pool (Fig 2A), where the angular unconformity between Strelley Pool Formation (dip 82^o^ south at 277^o^ on grid) and volcaniclastic sandstone of the Double Bar Formation (dip 68^o^ east at 160^o^ grid) is greatest [1]: a dip of 28^o^ east if Strelley Pool Formation is restored to horizontal. The Steer Ridge section was measured 2.6 km northwest across the North Shaw River from base camp on Antarctic Creek for the Trendall stromatolite locality [18], which in turn is 1 km northwest of the Trendall Ridge paleosol site studied here (Fig 2B) [16, 17, 33]. On Steer Ridge, conglomerate of the basal Strelley Pool Formation has a dip of 70^o^W on strike 329^o^ grid, and underlying chert in the Mt Ada Basalt is at 65^o^W on 330^o^ grid, for a restored dip of 3^o^ west. On Trendall Ridge, sandstone of the Strelley Pool Formation has a dip of 58^o^W on 329^o^ grid, and underlying ferruginous shales in the Panorama Formation is at 65^o^W on 339^o^ grid, for a restored dip of 4^o^ west. These low angular discordances are clear from some angles (Fig 2B), but have been misinterpreted as concordance [4]. The laminated ferruginous shales at Trendall Ridge (Fig 2F) are comparable with “jasper–siderite–Fe-chlorite banded iron formation” elsewhere in the Panorama Formation [35]. They lack pisolitic, boxwork, or massive textures of laterites [36], and so were not a deep weathering horizon 12–20 m within a very deep (25 m) pre-Strelley paleosol, as previously envisaged [4].

### Petrographic and geochemical study

A Swift automated stage linked to a Hacker electronic counting box were used to point count oriented thin sections prepared vertical to bedding for proportions of sand, silt, clay, and different mineral. Grains 5 μm in size and smaller were counted as clay, rather 2 μm, because pervasive silicification and recrystallization produced crystallites no less than 5 μm across [13],. Pyrophyllite books had distinctive with high birefringence, splayed and locally bent layers. Rock fragments were dominantly sedimentary, rarely metamorphic and basaltic. Evaporite minerals are now quartz pseudomorphs, with distinctive chalcedonic fabric. Original evaporite mineral determination followed previous identifications based on crystallography and electron microprobe analyses [6, 37, 38]. Opaque material counted includes both irregularly shaped kerogen and grains of opaque oxides. Two separate 500-point counts were made, one for grain size, and another for mineral composition of each sample (S1 and S2 Tables), with errors (2σ) of ± 2% for common (>8%) components [39].

Chemical analyses of selected samples were by ALS Chemex of Vancouver, British Columbia, using XRF on glass discs, and Pratt titration for FeO (S3 Table). The standard for the analyses was Canadian Certified Reference Materials Project standard SY-4, diorite gneiss from near Bancroft, Ontario, and 89 replicate analyses of the standard were used to calculate errors (2σ). Bulk densities were determined from 20–40 g samples by weighing raw and after paraffin coating, in and out of water [40] at the University of Oregon. Ten replicate density determinations of a single Western Australian Archean chert specimen (R4309) were used to calculate density errors. These data were used to calculate mass transfer and strain [41], concentration factors [42], and chemical index of alteration [43]. The relevant equations in Table 1 are the basis for calculating divergence from parent material composition.

**Table 1:** Transfer functions used to interpret Archean paleosols.

Equation	Variables		Coeffic-ient (R^2^)	Standard error	Refer-ence
$\tau_{j,w}=\left[\frac{\rho_{w}\cdot C_{j,w}}{\rho_{p}\cdot C_{j,p}}\right]\left[\varepsilon_{i,w}+1\right]-1$	*τ*_*w*,*j*_ (mole fraction) = mass transfer of a specified (j) element in a soil horizon (w); *ρ*_*w*_ (g.cm^-3^) = bulk density of the soil; *ρ*_*p*_ (g.cm^-3^ ) = bulk density of parent material; *C*_*j*,*w*_ (weight % ) = chemical concentration of an element (j) in a soil horizon (w); *C*_*p*,*w*_ (weight %) = chemical concentration of an element (j) in the parent material (p): ε_i,w_ (mole fraction) = strain due to soil formation		none	none	43
$\varepsilon_{i,w}=\left[\frac{\rho_{p}\cdot C_{i,p}}{\rho_{w}\cdot C_{i,w}}\right]-1$	*ε*_*i*,*w*_ (mole fraction) = strain of a soil horizon (w) with respect to a stable chemical constituent (i); *ρ*_*w*_ (g.cm^-3^) = bulk density of a soil horizon; *ρ*_*p*_ (g.cm^-3^) = bulk density of parent material; *C*_*i*,*w*_ (weight % ) = chemical concentration of stable element (i) in a soil horizon (w); *C*_*p*,*w*_ (weight %) = chemical concentration of stable element (i) in the parent material (p)		none	none	43
$CIA=\frac{100\left(mAl_{2}O_{3}\right)}{\left[\left(mAl_{2}O_{3}\right)+(mCaO)+\left(mNa_{2}O\right)+\left(mK_{2}O\right)\right]}$		*CIA* (ratio) = chemical index of alteration: *mAl*_*2*_*O*_*3*_ = moles alumina: *mCaO* = moles lime; *mNa*_*2*_*O* = moles soda*; mK*_*2*_*O* = moles potash (mole is weight percent divided by molecular weight).	none	none	45
$CO_{2}=\frac{M}{A[\frac{K_{CO_{2}}P}{1000}+\kappa \frac{D_{CO_{2}}\alpha }{L}]}$	*pCO*_*2*_ (atmospheres) = partial pressure of atmospheric carbon dioxide: *M* (mol CO_2_/cm_2_) = summed mass transfer losses of MgO, CaO, Na_2_O and K_2_O through the profile $M=2\sum \rho_{p}\frac{c_{j,p}}{100}\int_{Z=0}^{Z=D_{j,w}}\tau_{j,w(z)}\delta Z$; *ρ*_*p*_ (g.cm^-3^) = bulk density of parent material; *C*_*j*,*p*_(weight % ) = chemical concentration of an element (j) in parent material (p); *τ*_,*j*,*w*_ (mole fraction) = mass transfer of a specified (j) element in a soil horizon (w); *Z* (cm) = depth in soil represented by analysis; *A* (years) = duration of soil formation; $K_{CO_{2}}$ (mol./kg.bar) = Henry’s Law constant for CO_2_ (= 0.034, range 0.031–0.045); *P* (cm) = mean annual precipitation; *κ* (s.cm^3^/mol.year) = seconds per year divided by volume per mole of gas at standard temperature and pressure (= 1,409); $D_{CO_{2}}$(cm^2^/s) = diffusion constant for CO_2_ in air (= 0.162 at 20°C, range at 0–40°C of 0.139–0.181); α (fraction) = ratio of diffusion constant for CO_2_ in soil divided by diffusion constant for CO_2_ in air (= 0.1, range 0.08–0.12); *L* (cm) = original depth to water table (after decompaction, *B* below)				163
$O_{2}=\frac{F}{A\left.\frac{K_{O_{2}}P}{1000}+\kappa \frac{D_{O_{2}}\alpha }{L}\right]}$	*pO*_*2*_ *=* partial pressure of atmospheric oxygen (atmospheres); *F* (mol CO_2_/cm_2_) = summed mass transfer gains of Fe_2_O_3_ through the profile $M=2\sum \rho_{p}\frac{c_{j,p}}{100}\int_{Z=0}^{Z=D_{j,w}}\tau_{j,w(z)}\delta Z$; *ρ*_*p*_ (g.cm^-3^) = bulk density of parent material; *C*_*j*,*p*_(weight % ) = chemical concentration of an element (j) in parent material (p); *τ*_,*j*,*w*_ (mole fraction) = mass transfer of a specified (j) element in a soil horizon (w); *Z* (cm) = depth in soil represented by analysis; *A* (years) = duration of soil formation; $K_{O_{2}}$(mol./kg.bar) = Henry’s Law constant for O_2_ (= 0.00125, range 0.0012–0.0013); *P* (cm) = mean annual precipitation; κ (s.cm^3^/mol.year) = seconds per year divided by volume per mole of gas at standard temperature and pressure (= 1,409); $D_{O_{2}}$(cm^2^/s) = diffusion constant for O_2_ in air (= 0.203 at 20°C, range from 0–40°C is 0.179–0.227); α (fraction) = ratio of diffusion constant for O_2_ in soil divided by diffusion constant for O_2_ in air (= 0.2, range 0.09–0.32); *L* (cm) = original depth to water table (after decompaction, *B* below)				28
$A=\frac{N\cdot 1,000,000}{4470}$	*A* (yrs) = duration of soil formation; *N* (cm) = total profile thickness		0.99	±24,608 yrs	131
*A* = 465.31·*N* -21358	*A* (yrs) = duration of soil formation; *N* (cm) = total profile thickness		0.94	±23,192 yrs	134
*A* = 1037.3·*N* -14202	*A* (yrs) = duration of soil formation; *N* (cm) = total profile thickness		0.86	±5,486 yrs	133
*A* = 6712.5·*N* -39622	*A* (yrs) = duration of soil formation; *Y* = total profile clay (g.cm^-2^)		0.87	±112,819 yrs	132
*A* = 5061.1·*H* -438008	*A* (yrs) = duration of soil formation; *H* = solum thickness (cm)		0.60	±190,883 yrs	132
*A = 3920·S* ^*0*.*34*^	*A* (yrs) = duration of soil formation; *S* = diameter of micritic low-magnesium calcite nodules (cm^-^)		0.57	±1,800 myrs	129
*A = 3987·G + 5774*	*A* (yrs) = duration of soil formation; *G* = proportion of surface covered by gypsum crystals or nodules (%)		0.95	±15,000 yrs	27
*P* = 221e^0.0197*R*^	*P* (mm) = mean annual precipitation; *R* (moles) = 100mAl_2_O_3_/(mAl_2_O_3_+mCaO+mNa_2_O)		0.72	±182 mm	139
*T* = 0.21*I –*8.93	*T* (^o^C) = mean annual palaeotemperature; *I* (mole fraction) = chemical index of weathering $\left(I=\frac{100\cdot Al_{2}O_{3}}{\left(Al_{2}O_{3}+CaO+Na_{2}O\right)}\right)$		0.81	±0.5°C	141

Thin sections and their source rocks are curated in the Condon Collection of the Museum of Natural and Cultural History of the University of Oregon, in Eugene, Oregon. Microfossils also were examined using a FEI Helios Dual Beam, Focused Ion Beam Microscope for scanning electron microscopy in the Center for Advanced Materials Characterization at Oregon (CAMCOR) of the University of Oregon.

### Detrital zircon dating

A rock sample from 46 m in the section at Marble Bar (Fig 3D: R3809 in Museum of Natural and Cultural History of the University of Oregon) was crushed to liberate zircons for determination of U-Pb ages [44]. Small (100–200 μm), equant to prismatic, euhedral to subhedral zircon crystals were separated by density and magnetism in the Isotope Geology Laboratory of Boise State University by magnetic drum separation. The zircon separate was placed in a muffle furnace at 900°C for 60 hours in quartz beakers in order to anneal minor radiation damage.

Laser ablation ICPMS analysis utilized a ThermoFisher X-Series II+ quadrupole inductively coupled plasma mass spectrometer coupled to New Wave UP213 UV laser ablation system. Zircon was ablated with a laser spot of 20 μm wide using fluence and pulse rates of ∼2 J.cm^-2^ and 5 Hz. A 45 second analysis (15 sec gas blank, 30 sec ablation) excavated a pit ∼10 μm deep. Ablated material was carried by a combined 1.2 L.min^-1^ He gas stream from the two-volume ablation cell to the nebulizer flow of the plasma. Quadrupole dwell times were 5 ms for Si and Zr, 200 ms for ^49^Ti and ^207^Pb, 80 ms for ^206^Pb, 40 ms for ^202^Hg, ^204^Pb, ^208^Pb, ^232^Th, and ^238^U and 10 ms for all other high field strength elements and rare earth elements; total sweep duration was 950 ms. Background count rates for each analyte were obtained prior to each spot analysis and subtracted from the raw count rate for each analyte. For concentration calculations, background-subtracted count rates for each analyte were internally normalized to ^29^Si and calibrated with respect to NIST SRM-610 and -612 glasses as the primary standards. Ablation pits that intersected glass or mineral inclusions were identified based on Ti and P signal excursions, and associated sweeps were generally discarded. Signals at mass 204 were normally indistinguishable from zero following subtraction of mercury backgrounds measured during the gas blank (<100 cps 202Hg), and thus dates are reported without common Pb correction. Rare analyses that appear contaminated by common Pb were rejected.

For U-Pb and ^207^Pb/^206^Pb dates, instrumental fractionation of the background-subtracted ratios was corrected and dates were calibrated by interspersed measurements of zircon standards. Plešovice zircon standard was used to monitor time-dependent instrumental fractionation, with two analyses for every 10 analyses of unknown zircons. Radiogenic isotope ratio and age error propagation for all analyses includes uncertainty contributions from counting statistics and background subtraction. Isotopic dilution TIMS analysis used crystals previously mounted, polished and imaged by cathodoluminence (CL), and analyzed by laser ablation ICPMS. Single crystal fragments plucked from grain mounts were individually abraded with concentrated HF at 180°C for 12 hours. Chemically abraded crystals were spiked with the EARTHTIME ET535 traced, totally dissolved, Pb and U purified by anion exchange chromatography, and their isotope ratios measured on an IsotopX Isoprobe-T thermal ionization mass spectrometer in single collector, Daly detector (Pb), or static multicollector Faraday cup (U) routines, Pb and U purified by ion exchange. U-Pb dates and uncertainties for each analysis were calculated using established algorithms [44]. All data for ^207^Pb/^206^Pb dates are in S4-S7 Tables- within a single Excel file.

## Newly recognized Strelley Pool Formation near Marble Bar

### New geological mapping

**Fig 6:**
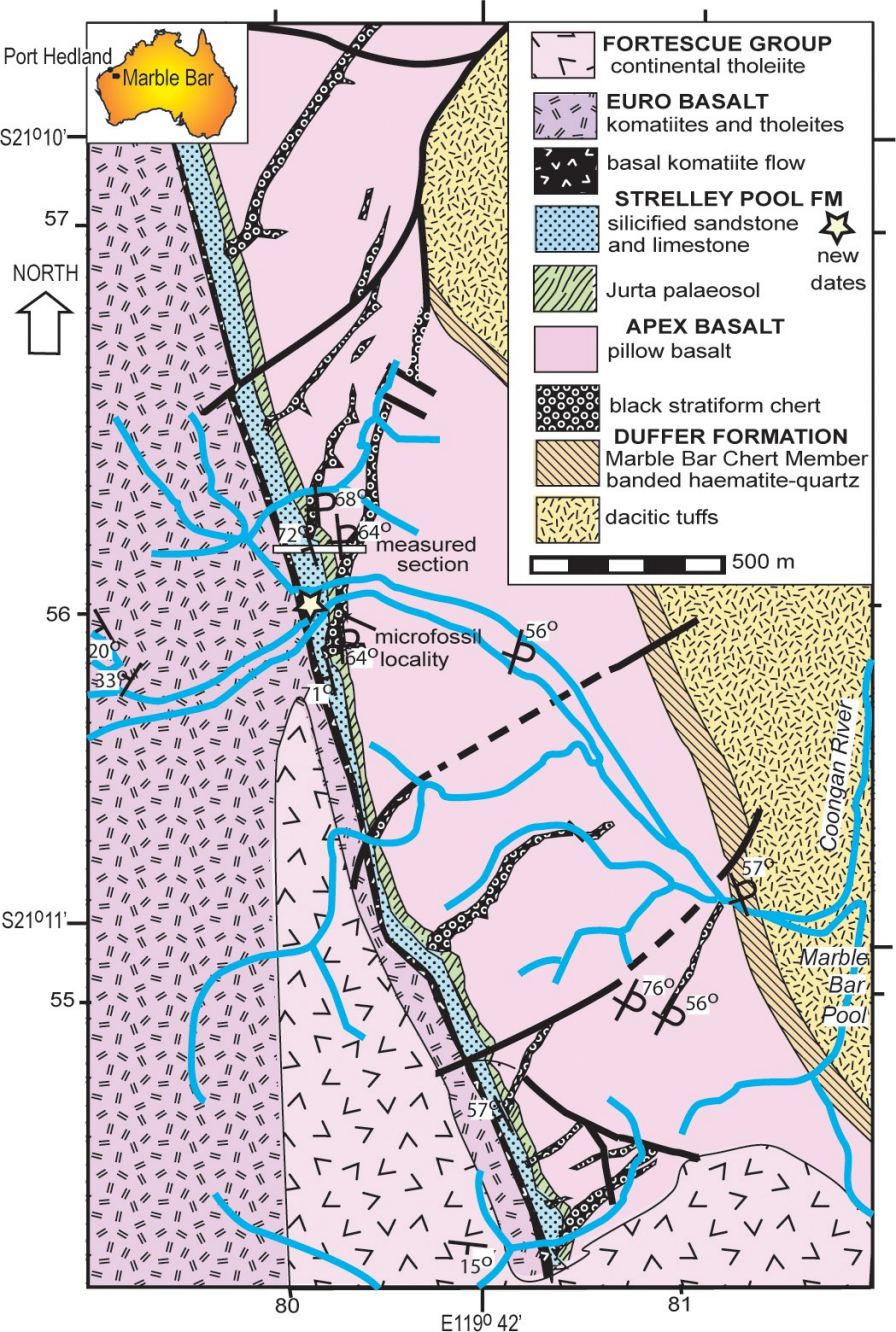
Geological map of creeks west of Marble Bar Pool, with a new interpretation of formations there. The Jurta paleosol, Strelley Pool Formation and Euro Basalt were mapped previously as Apex Basalt [7, 46, 47, 49].

A surprise of this work was discovery that Strelley Pool Formation and the pre-Strelley paleosol at well known localities (Fig 3A–3C) were identical to a **white chert** and its underlying paleosol currently mapped [6, 9, 45, 46] as Apex Formation near Marble Bar (Fig 3D; S21.17603^o^ E119.69779^o^), and directly above the fossiliferous **black chert** [10] of the Apex Formation (white asterisk in Fig 2C). Five reasons for revised geological mapping of this small area (Fig 6) are given in the following paragraphs. The distribution of lithologies is not in dispute, only their stratigraphic relationships.

First, the microfossiliferous black chert in the Apex Formation [10] does not taper to the east [6], but remains 15 m thick across the valley, as later mapped [7, 9, 47]. The black chert dips 64^o^ east at 172^o^ on grid (Fig 2C) conformable with basalt in the creek nearby (S21.17597^o^ E119.70175^o^), which dips 59^o^ east at 175^o^ grid (Fig 2D): these are at restored angles of 8^o^ and 12^o^ (respectively) to the bedded white chert forming the top of the long strike-ridge, which dips 71^o^ west at 158^o^ grid (Fig 5B). This low-angle restored geometry is incompatible with the idea that the black chert filled a vertical dilational crack [6, 7, 45, 48]. The black chert does not branch and widen vertically upwards and anastomose like proven hydrothermal dikes cutting the Duffer Formation [48], but is conformable with pillow basalts and interbedded cherts within the sequence (Fig 6).

Second, the black microfossiliferous chert has ripple marks, rounded rip-up clasts, and normally graded laminae [10], conformable with (Fig 2C), rather than cutting across, interbedded pillow basalts (Fig 2D). Very mild thermal alteration of the black chert is indicated by a modest europium anomaly [47], and a low-temperature (80–150°C) mineral suite of halloysite, alunite, goethite, and jarosite [38]. No stockwork style brecciation, nor sulfide ore minerals were seen as would be expected for hydrothermal hot spring interpretation [6, 7, 45]. Nor are there slickensides, nor offset of the ridgeline white chert, to encourage the idea that these black cherts are emplaced along major faults.

**Fig 7:**
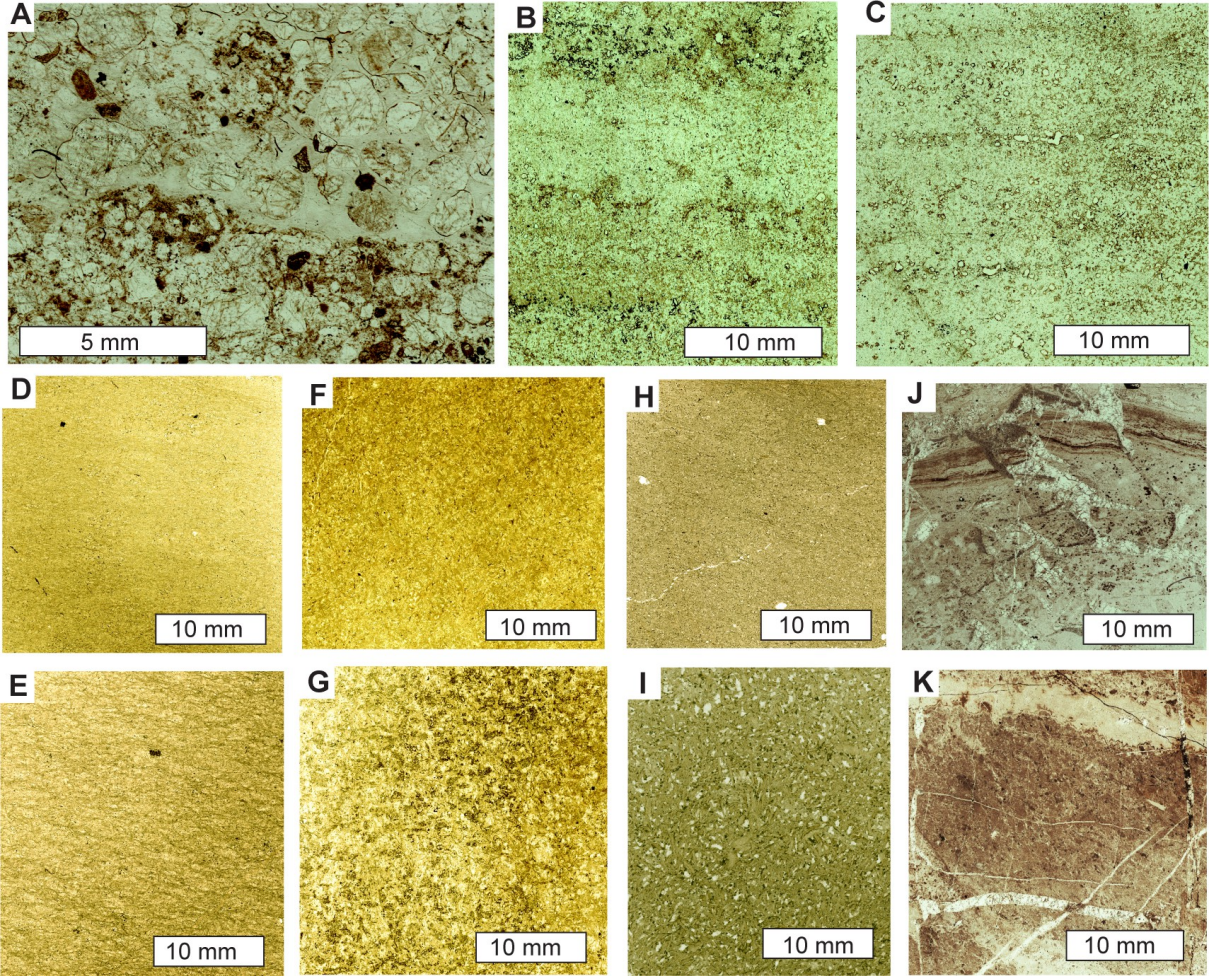
Petrographic thin sections of sediments of the Strelley Pool Formation and paleosols and their protoliths of the underlying unconformity in plane-polarized light, showing massive pedogenic rather than bedded fabrics: **A**, basal Strelley Pool Formation with brown clasts of underlying Jurta paleosol at Strelley Pool; **B**, disrupted bedding in A horizon of Jurnpa loam paleosol at Trendall Ridge; **C**, bedded C horizon of Jurnpa loam paleosol in Trendall Ridge; **D**, A horizon Jurta clay paleosol at Trendall Ridge; **E**, R horizon Jurta paleosol of felsic metatuff of Panorama Formation at Trendall Ridge; **F**, A horizon Jurta clay loam paleosol at Strelley Pool; **G**, R horizon of Jurta clay loam paleosol of metabasalt of Double Bar Formation at Strelley Pool; **H**, A horizon of Jurta silty clay loam paleosol near Marble Bar; **I**, R horizon of Jurta silty clay loam paleosol of metabasalt of Apex Basalt near Marble Bar; **J**, C horizon of Wanta loam paleosol near Marble Bar; **K**, Green tuffaceous siltstone with replacive caliche vein (upper part) near Marble Bar. Thin sections and specimen numbers in the Museum of Natural and Cultural History of the University of Oregon are R4202 (A), R4321 (B), R4323 (C), R4504 (D), R4507 (E), R4204 (F), R4206 (G), R3784 (H), R3789 (I), R3794 (J) and R3809 (K).

Third, black chert beds within the Apex Basalt protrude upwards from an angular unconformity with the white ridgeline chert (Fig 5B). The basaltic portions below this unconformity were paleotopographically lower parts of a 4-m-thick sericite mapped intermittently for 4 km along strike (as unit AWAa-fnt [49]). This thick paleosol is rich in pyrophyllite (Fig 7H and 7I) like the pre-Strelley paleosol elsewhere (Fig 7D–7G), due to regional hydrothermal alteration associated with domal uplift creating the angular unconformity [3]. No dispersed pyrophyllite, only small pyrophyllite casts, were found in the ridge-capping white cherts, overlying green tuffs, basalts, and komatiite (Fig 7B and 7C and 7J). Nor do the white ridgeline cherts have a europium anomaly [47], nor negative δ^30^Si values [50], nor halloysite-alunite-goethite-jarosite hydrothermal alteration [38] of the black chert. The contact between the black chert and white chert of the ridge top has been accurately mapped before [7, 9], showing black chert protruding upwards into a brecciated top and surrounded by flanking black clasts in a matrix of white chert. This map distribution is like a fractured outcrop of chert with flanking scree on the ancient landscape of the angular unfconformity, rather than spiracles, chimneys, rim-pools, boxworks, or interdigitation of hydrothermal vents and flanking deposits.

Fourth, the sequence of facies within the white ridgeline chert are identical with those of Strelley Pool Formation elsewhere (Fig 5). Near Marble Bar, this begins with (1) locally conglomeratic quartz sandstone, then (2) chert with evaporites, (3) banded chert with microbial lamination and rollups, (4) intraformational conglomerate, and (5) green tuffacous siltstone (Fig 7K), like all other outcrops of Strelley Pool Formation [18, 19, 51]. Silicified microbial carbonates like those elsewhere in the Strelley Pool Formation [21–25], have also been documented near Marble Bar [9]. The overlying komatiite and then basalt lack hydrothermal alteration like Euro Basalt, and unlike Apex Basalt [52]. Felsic rocks previously mapped as Panorama Formation 1 km up-section to the west [46, 49] are green-gray and massive like felsic tuffs within the Euro Basalt and Leilira Formation [28], but unlike grey to red Panorama Formation in the North Pole Dome (Fig 2E).

Fifth, this newly recognized outcrop of Strelley Pool Formation (Fig 6), fills a conspicuous gap in the strike ridge of white chert around the Corunna Downs and Mt Edgar granitic complexes (Fig 1). Near Blue Bar of the Coongan River (S21.409888^o^ E119.733383^o^) south of Marble Bar Pool, Strelley Pool Formation is unconformable on Panorama Formation and conformably overlain by Euro Basalt [46]. In Doolena Gap (S20.929091^o^ E119.784748^o^) to the north, Strelley Pool Formation is unusually thick (1000 m) and has eroded down through all the Apex Basalt and Marble Bar Chert into Duffer Formation, but again is conformably overlain by Euro Basalt [53]. At Kittys Gap to the east (S20.885075^o^ E120.072608^o^), “Kittys Gap chert” is now regarded as Strelley Pool Formation [46], and is unconformable on Panorama Formation over Apex Basalt, and again conformably overlain by Euro Basalt [54]. Previous mapping [6–9, 45, 46] assumes that the Panorama and Strelley Pool Formation are buried beneath Fortescue Basalt 2–3 km to the west, and requires unsupported block faulting of at least that magnitude.

The new interpretation offered here is that this locality shows white cherty Strelley Pool Formation, unconformable on Apex Basalt with black chert interbeds, and overlain by basal komatiite of Euro Basalt (Fig 6). By this interpretation the Apex Basalt onlaps the Marble Bar Chert Member of the Duffer Formation (Fig 4), which is known to have been deformed during caldera collapse before Apex Basalt flows [55]. This new interpretation does not contradict past lithological mapping, but does contradict the idea that both black hillside and white ridgeline cherts were entirely within the Apex Basalt [6–9, 45, 46]. Fundamental to the new interpretation is recognition of the same 4 m thick sericitic paleosol and angular unconformity first recognized elsewhere in the Pilbara region [1], as well as similarities in facies and paleosols within the white cherts throughout East Pibara Shire (Fig 3).

### New detrital zircon dating

Green siltstone at S21.175438^o^ E119.697185^o^ (at star in Fig 6), and at 43 m within the stratigraphic section measured near Marble Bar (Fig 4E) is poorly exposed only in the creek bed. The siltstone (sample R3809) has planar bedding and dips steeply west like the surrounding white cherts, but is broken into a boxwork and cemented by partially replacive calcrete (Fig 7K). This siltstone has been misinterpreted as an airfall volcanic tuff [6], but is a volcaniclastic siltstone with clear bedding and scattered shard-like quartz grains (Fig 7K).

**Fig 8:**
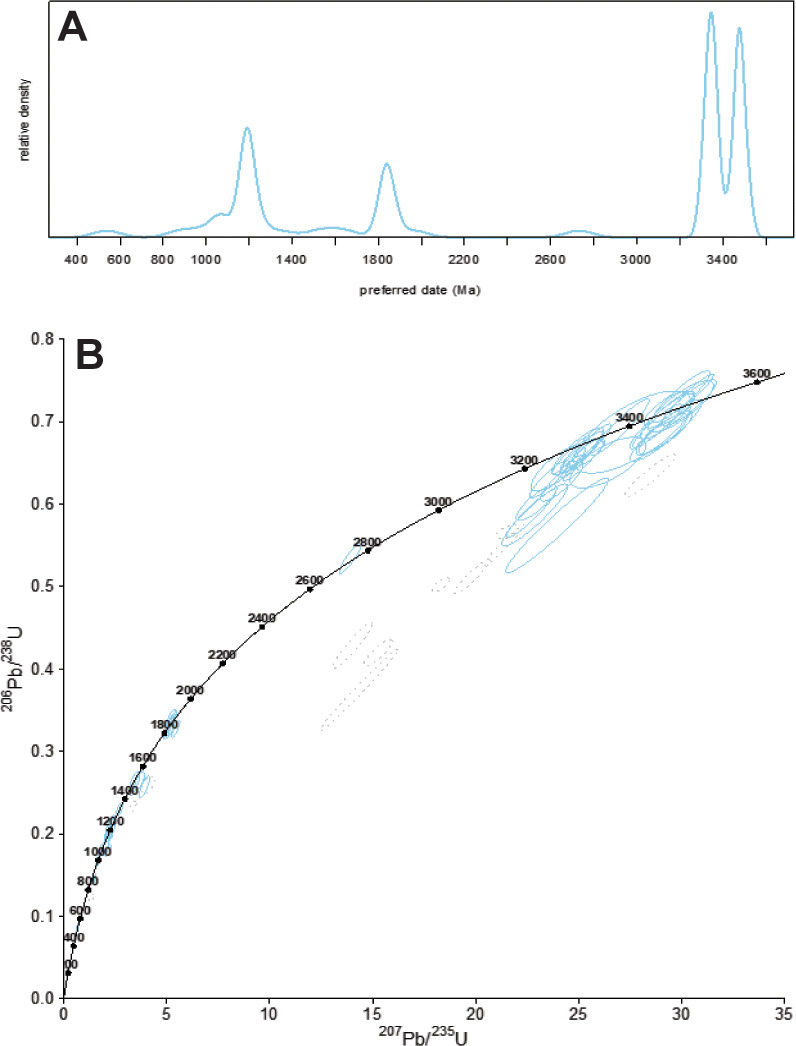
Detrital zircon ages from sample R3809 of upper Strelley Pool Formation near Marble Bar: A, Kernel density estimate diagram for LA-ICPMS spot dates: B, Concordia diagram of LA-ICPMS spot dates.

The 65 individual detrital zircon crystals from R3809 analyzed by LA-ICPMS yielded a heterogeneous age spectrum with spot dates ranging from 3595 ± 19 Ma to 539 ± 23 Ma, including dominant modes at 1200 (n = 9), 1850 (n = 6), 3350 (n = 15), and 3450 (n = 14) Ma, amongst the analyses passing a ≤5% discordance criterion (Fig 8). Nine crystals from the *ca* 3350 Ma mode of LA-ICPMS data were plucked from the epoxy mounts and analyzed by CA-IDTIMS methods. These crystals provided variable discordant U-Pb isotope ratios (Fig 8A); four near concordant analyses define a weighted mean ^207^Pb/^206^Pb date of 3316.0 ± 0.6 Ma (n = 4; MSWD = 0.17), and including three more discordant grains, define an upper intercept date of 3316.2 ± 0.4 Ma (n = 7; MSWD = 0.25). The remaining crystals yielded resolvably younger and concordant to slightly discordant analyses with ^207^Pb/^206^Pb dates of 3310.1 ± 0.7 Ma and 3281.6 ± 0.9 Ma. Four crystals were selected from the *ca* 3450 Ma mode of the LA-ICPMS data for analysis by CA-IDTIMS. These crystals exhibit variably discordant U-Pb isotope ratios (Fig 8B), but define a statistically significant weighted mean ^207^Pb/^206^Pb date of 3466.7 ± 0.4 Ma (n = 4; MSWD = 2.14), and an upper intercept date of 3466.2 ± 0.6 Ma (n = 4; MSWD = 1.3).

**Fig 9:**
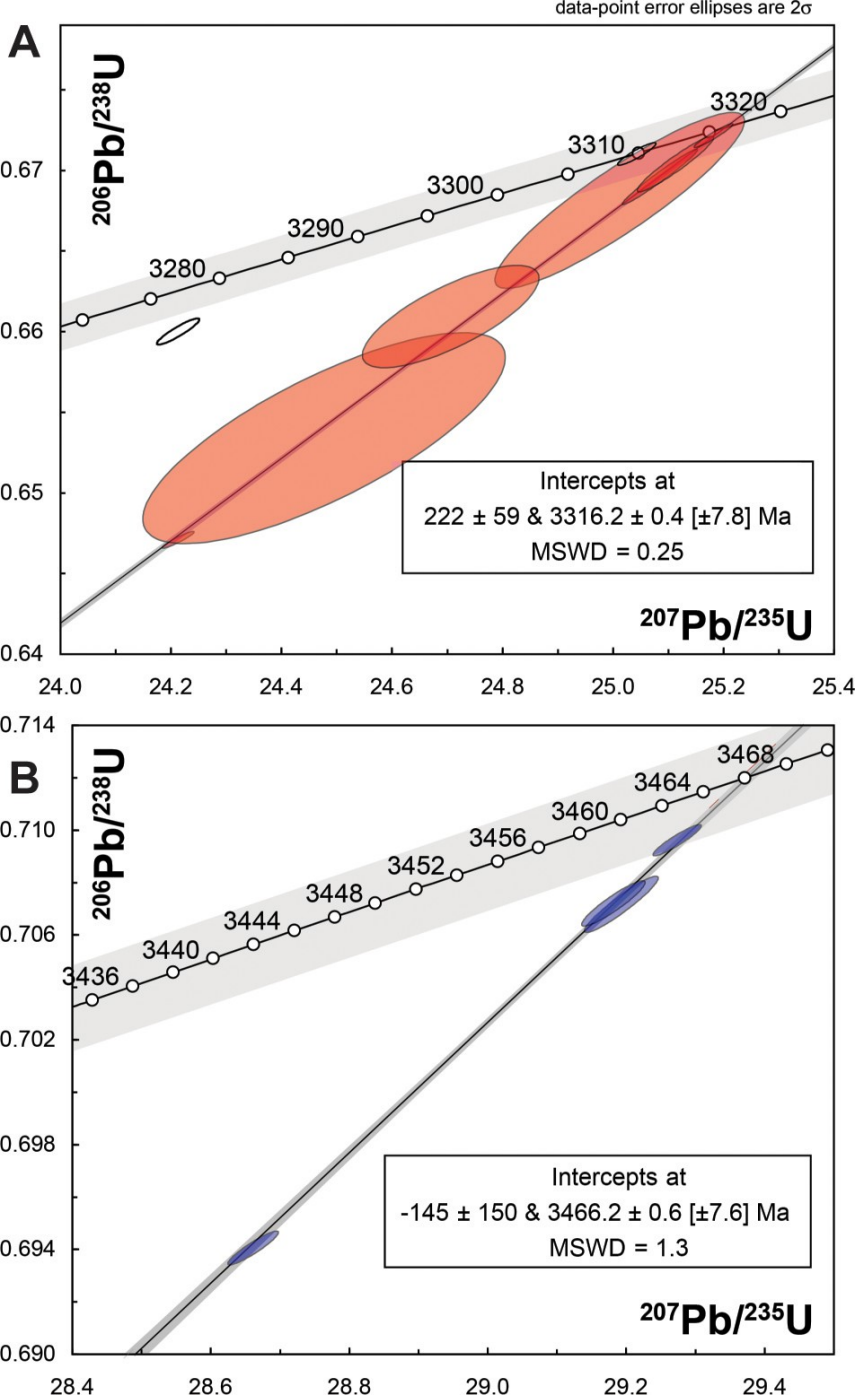
New U-Pb dates on detrital zircons from sample R3809 taken to represent the age of the Strelley Pool Formation (A) and Apex Basalt (B). These are CA-IDTIMS dates of selected grains in the two Archean modes identified by LA-ICPMS spot analyses.

The scatter of radiometric dates demonstrates that the sampled rock has a compendium of detrital zircons, mostly of two distinct geological ages: 3316.2 ± 0.4 Ma and 3466.2± 0.6 Ma (Fig 9). One or two zircons each represent the Fortescue Group which is a large igneous province dated at about 2735 Ma [56], Ashburton Formation representing Capricorn Orogeny at about 1827 Ma [57], Wankanki Granites of Mount West Orogeny at about 1335 Ma [58], Pintjantjatjara Granites of the Musgrave Orogeny at about 1197 Ma [58], and Rudall Metamorphic Complex of Alice Springs Orogeny at about 539 Ma [59]. These younger detrital zircons are interpreted as contaminants from Cenozoic calcrete in cracks cementing the specimen (Fig 7K), because they are so much younger than any other rocks within the mapped area (Figs 1 and 2). Similar contamination with zircons dated from 550–2700 Ma has been found in bedrock dated to 3100–3050 Ma in the Jack Hills, Western Australia [60]. Single grains with Proterozoic to Phanerozoic ages delivered as dust to cracks in bedrock over a long period of time widely contaminate this stable continental landscape, so we do not consider ages based on single grains to be significant.

**Fig 10:**
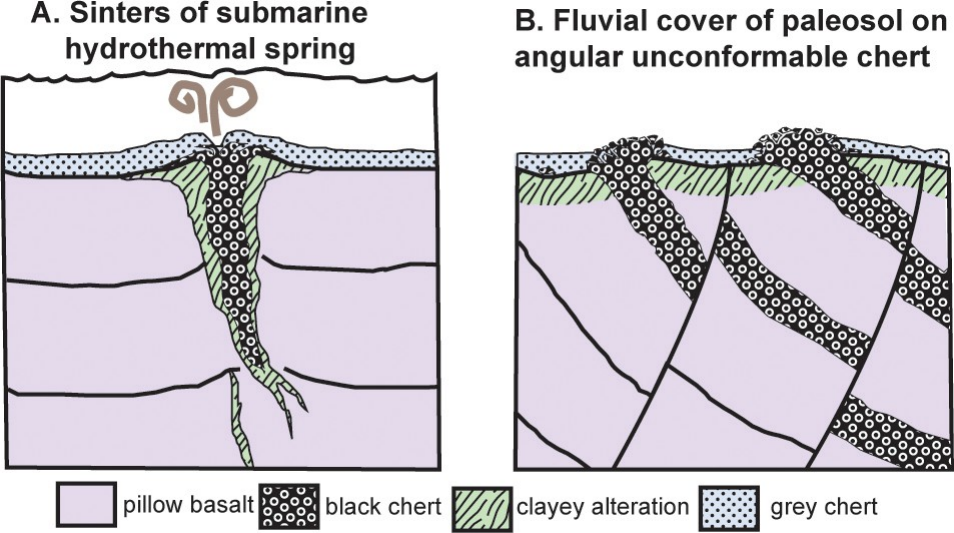
Alternative scenarios for the Archean Apex chert microfossil locality, as a shallow submarine hydrothermal vent (A) or a deformed marine bed at an angular unconformity (B).

Our date of 3316.2 ± 0.4 Ma for the maximum depositional age of the siltstone conformable within the ridge-forming white chert is most compatible with ages for overlying dikes, dacites and rhyolites of the Kelly Group large igneous province [61], initiated with volcanic breccias and paraconformities during deposition of the upper Strelley Pool Formation [46, 62, 63]. It is comparable with maximal depositional age of 3350 ± 3 Ma for zircons at Strelley Pool near the base of the Euro Basalt [33]. Our other mode of 3466.2 ± 0.6 Ma is most like ages of dacites and rhyolites of the nearby Duffer Formation (Figs 1 and 6), which is a likely source of those zircons (Fig 2). Thus, the white ridge-forming chert is interpreted as Strelley Pool Formation with maximum deposition age of 3316.2 ± 0.4 Ma, and at least a 143 million-year duration for the angular unconformity between the Strelley Pool Formation and Apex Basalt at 3459 ± 2 Ma [54]. In areas with intervening Panorama Formation dated at 3446 ± 5 Ma [54], the hiatus was at least 130 Ma. This geological and radiometric evidence for a major unconformity (Fig 10B), as elsewhere in the region [1], falsifies submarine hydrothermal vent interpretation [6–9] of these rocks (Fig 10A).

## Diagenetic and metamorphic alteration

### Metamorphism

This study aims to reconstruct ancient soil formation, itself is a form of early diagenetic alteration: after deposition or erosion, but before burial. It is vital to separate soil formation from post-burial, late diagenetic, hydrothermal, and metamorphic alteration. The Strelley Pool Formation was altered by chlorite-calcite-epidote lower greenschist metamorphism like the overlying Euro Basalt [2]. Metamorphism of the underlying Panorama Formation was within the upper greenschist facies (ca. 350°C [64]). Pyrophyllite hydrothermal alteration at some 100–350°C of Apex and Mt Ada Basalts and Panorama Formation unconformably underlying the Strelley Pool Formation is linked to intrusion of the North Pole Monzogranite and mapped [3] throughout the study area (Fig 1). Lower temperature (80–150°C) assemblages of halloysite-goethite-jarosite-alunite [38] may have been protected by early diagenetic silicification of the Apex chert near Marble Bar [10], unlike high temperature pyrophyllitic alteration of enclosing Apex Basalt [3]. Raman spectroscopic ratios (D1/(D1+G)) of organic matter is evidence of temperatures: about 400°C for the Panorama Formation, but only 200°C for the middle Strelley Pool Formation, and 300–400°C for the upper Strelley Pool Formation [65]. The middle Strelley Pool Formation organic matter may also have been protected by early diagenetic silicification, as it has not been graphitized, and retained large polycyclic aromatic units [66]. Regional hydrothermal alteration predates formation of the pre-Strelley paleosol [1], because pyrophyllitic paleosol clasts are incorporated in basal conglomerates of the Strelley Pool Formation (Fig 7A [18, 19]), and because pyrophyllitic alteration is much deeper below the Strelley Pool Formation than the underlying sericitic paleosol [4].

Greenschist facies regional metamorphism is compatible with likely depth of burial of the Strelley Pool Formation. Around North Pole Dome, the Strelley Pool Formation is 28 m thick, Euro Basalt 9,400 m, Sulfur Springs Group 4,941 m [28], Gorge Creek Group 2,610 m [48], for a total of 16,980 m overburden. Near Strelley Pool, the thickness of Euro Basalt is only 1,000 m [1], so overburden below Lalla Rookh and Fortescue Groups would have been 8,580 m. The Lalla Rookh Formation of the overlying De Grey Group was confined to fault basins [67], and its 3,000 m did not overlie the studied localities. Fortescue Group basalts and sediments northwest of Marble Bar are 5,240 m thick [53, 68], for a total burial depth of about 13,826 m.

### Late diagenesis

Alunite is a common diagenetic mineral [69], and was noted during our study in veins associated with brittle deformation of the Strelley Pool Formation. Alunite has been linked to continuing hydrothermal alteration in Archean rocks of Western Australia [2], but alunite is not always a magmatic-hydrothermal or magmatic-steam mineral [70]. Alunite used for radiometric dating of unburied or non-hydrothermal Cenozoic paleosols in Australia [71] was formed by acid-sulphate weathering less than 30 m from the land surface [72]. Cenozoic paleosol alunite is cryptocrystalline, and also forms euhedral crystals 5 μm across [73].

**Fig 11:**
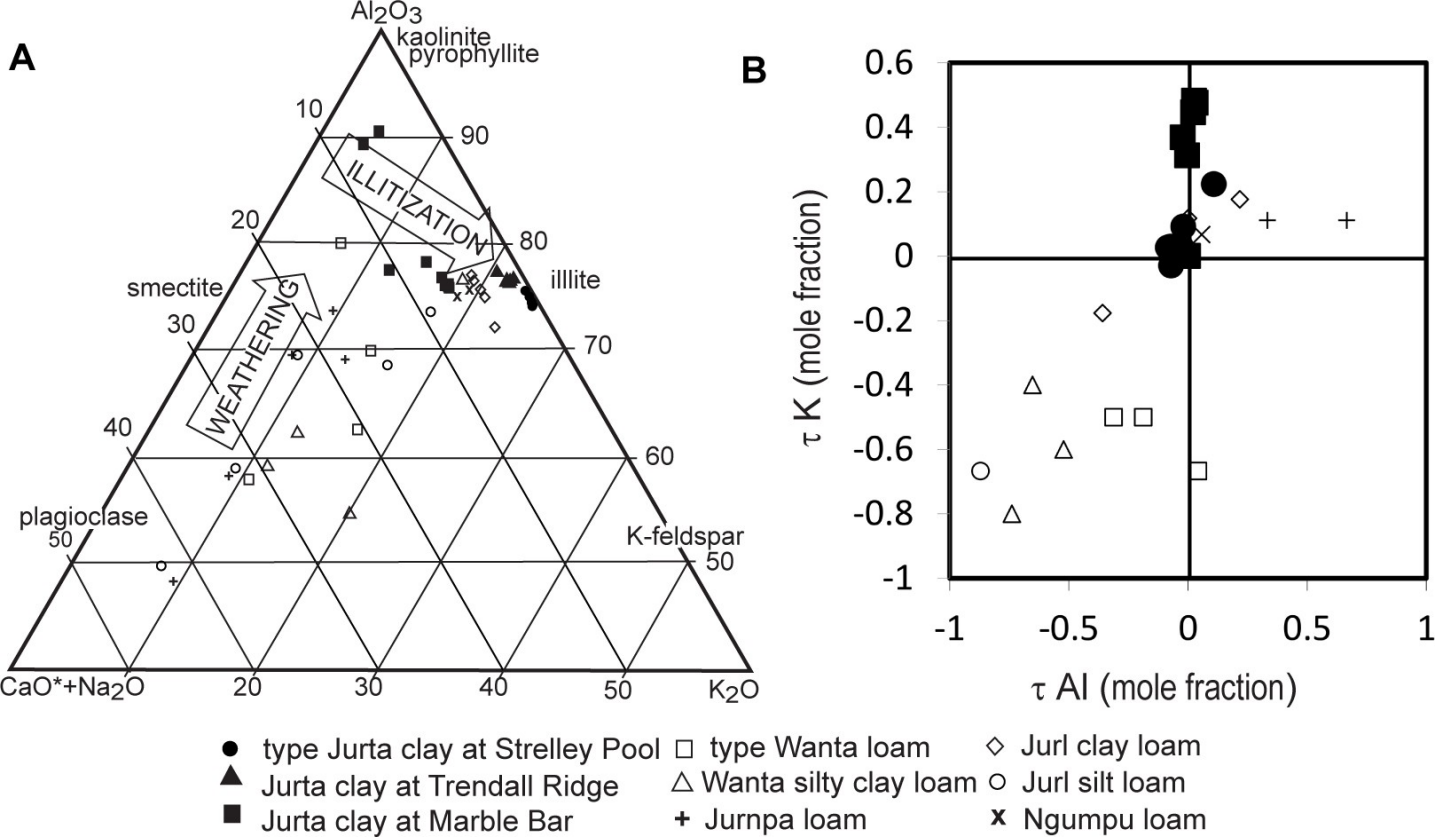
Illitization of paleosols within (open symbols) and below (closed symbols) the Strelley Pool Formation, showing that some profiles have been altered by illitization during deep burial, but most have not. For comparable illitized paleosols and unillitized soils see [75].

Illite may be another indication of deep burial alteration diverting weathering trends from culmination in kaolinite [43, 74, 75]. The observed trends in the Strelley Pool Formation and its underlying paleosol are more pronounced (Fig 11) than expected from less severe weathering of potassium from soils before the advent of land plants [76, 77]. Illitization has not affected paleosols within the Strelley Pool Formation, which were protected by silicification predating deep burial [46, 63, 78]. Parts of the unsilicified pre-Strelley paleosols were demonstrably affected by illitization, but only by a mole fraction, compared with 8–12 moles seen in 1.9 Ga Drakenstein and Shreiber Beach paleosols [77].

### Early diagenetic silicification

**Fig 12:**
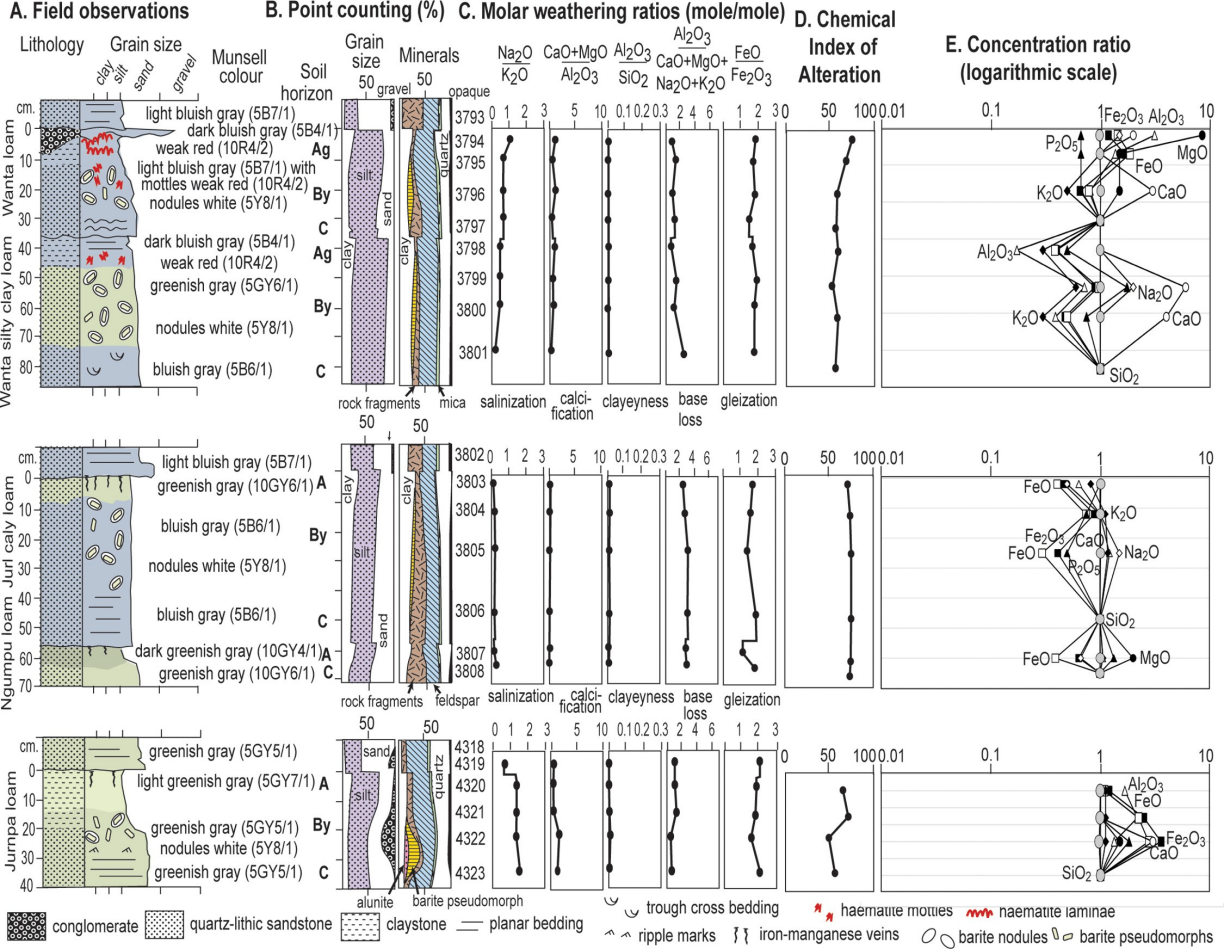
Alluvial paleosols within the Strelley Pool Formation, with petrographic and chemical data, Chemical Alteration Index [43] and chemical concentration ratios [42]. Early diagenetic silicification has replaced common sand crystals and nodules of sulphates and preserved weathering trends comparable with those of deeply weathered pre-Strelley Jurta paleosols (Fig 13).

**Fig 13:**
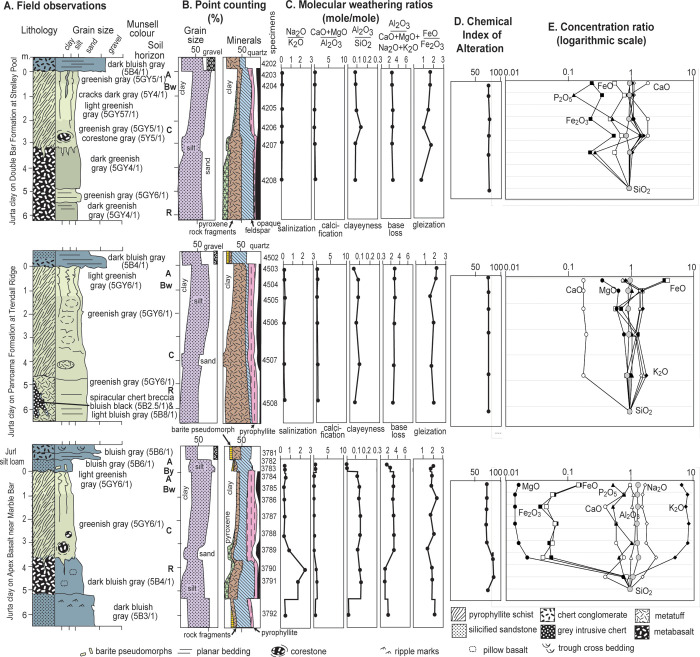
Jurta paleosols at the angular unconformity beneath the Strelley Pool Formation, with petrographic and chemical data, Chemical Alteration Index [43] and chemical concentration ratios [42]. These deeply weathered paleosols formed on a variety of bedrock basalts and felsic tuffs.

A special problem for Archean fossils is silicification, which has replaced stromatolitic dolostones, sandstones, and conglomerate of the Strelley Pool Formation [18, 19]. Beds within the Strelley Pool Formation show little difference in silicification between different parts of the same beds (Fig 12), but the thick pre-Strelley paleosols have much less silica, and show mild enrichment in silica up-profile (Fig 13). Such distributions favor an early-diagenetic post-burial rather than pedogenic origin of silicification.

One possibility is hydrothermal silicification related to chert dikes and pyrophyllitic alteration associated with intrusion of the North Pole Monzogranite, or a paleovolcano in the Panorama Formation, or emplacement of tuffs and flood basalts of the Euro Basalt [38, 47, 48]. Arguing against hydrothermal silicification of the Strelley Pool Formation, Hickman [38] emphasized the tens-of-million-year age gap between basement rocks and Strelley Pool Formation, and redeposition of pyrophyllitic clasts of basement rocks into basal conglomerates of the Strelley Pool Formation (Fig 7A). To this can be added the vast extent of Strelley Pool Formation (Fig 1), well beyond the hydrothermal cupola of North Pole Monzogranite [3]. Analyses of rare earth elements from the Strelley Pool Formation fail to show the positive europium anomaly of other Archean cherts considered hydrothermal [35, 63, 79, 80]. Fluid inclusion studies of the stromatolitic cherts reveal silica precipitation from waters low in salinity (< 3 wt % NaCl) and cool (<120°C) for hydrothermal solutions [81]. Additional evidence against hydrothermal silicification is positive δ^30^Si isotopic compositions of “Kittys Gap Chert” [50, 82–84], regarded as Strelley Pool Formation [38]. In contrast, negative δ^30^Si values of Cleaverville, Point Samson, Apex and Marble Bar Cherts are considered hydrothermal [50]. Finally, the 4-m-thick unsilicified paleosol underlying the Strelley Pool Formation separates strongly silicified Strelley Pool Formation from silicified tuffs of the Panorama Formation and Double Bar, Mt Ada and Apex Basalts (Fig 3). Thus, Strelley Pool Formation postdated, and was not affected by deep hydrothermal fluids producing pyrophyllite.

Another possibility is that cherts of the Strelley Pool Formation are silcretes formed by Cenozoic weathering, as suggested [28] from casual observations that silicification is more common on ridges than in valleys with unaltered stromatolitic ferroan dolostones of the Strelley Pool Formation. This is not a compelling argument, because creeks may have preferentially followed carbonates rather than cherts, which remain weather-resistant (Fig 2). The cherts are unlikely to be Cenozoic for four reasons. First, chert of the Strelley Pool Formation was found at depths of 142.6 to 163.6 m in Coonterunah core no. 8 of the Archean Biosphere Drilling Project [85]. Second, Strelley Pool cherts show pervasive neomorphic recrystallization to domains at least 5 μm in size requiring deep burial [13]. Third, the uppermost Strelley Pool Formation includes breccias with fragments of both chert and limestone redeposited from older parts of the formation [19]. Fourth, Australian Cenozoic silcretes are different chemically: they have more titania (0.7–48.0 weight % [86, 87]) than cherts of the Strelley Pool Formation (0.02–1.18 wt %: S3 Table). Cenozoic silcretes also have negative δ^30^Si values [88], whereas δ^30^Si values of Strelley Pool Formation are positive [50].

**Fig 14:**
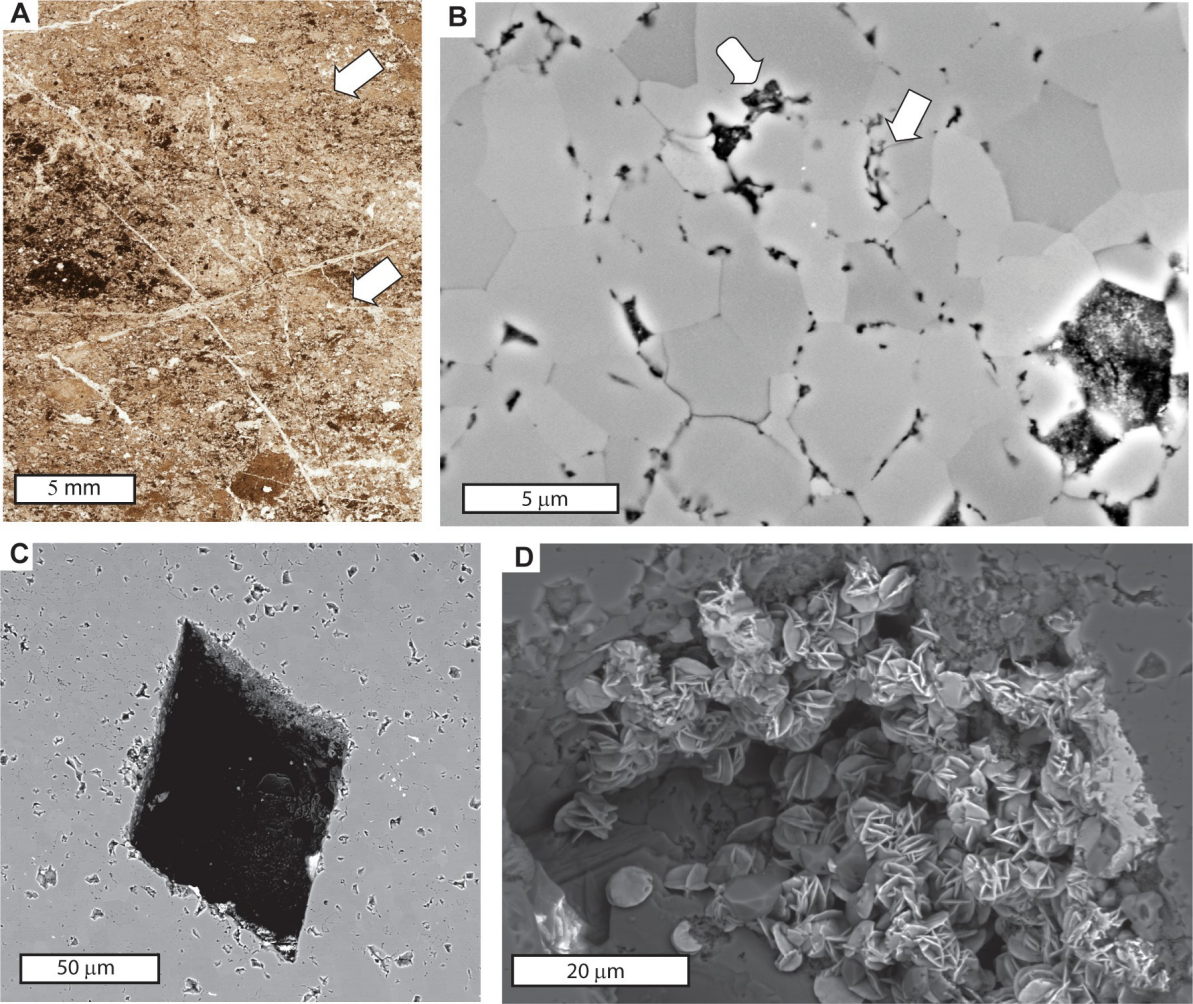
Minerals and organic matter of the microfossil locality of Schopf and Packer (1987) in chert of Apex Basalt near Marble Bar: **A**, petrographic thin section in plane polarized light of microfossiliferous rounded black chert clasts in graded beds (contacts at arrows); **B**, back-scatter scanning electron micrograph showing microfossils at arrows resisting tendency of exclusion by neomorphic recrystallization; **C**, back-scatter scanning electron micrograph of barite external mould; **D**, secondary mode scanning electron micrograph of rosettes of hematite in external mould of barite. All images are from specimen R4201B in the Museum of Natural and Cultural History of the University of Oregon.

**Fig 15:**
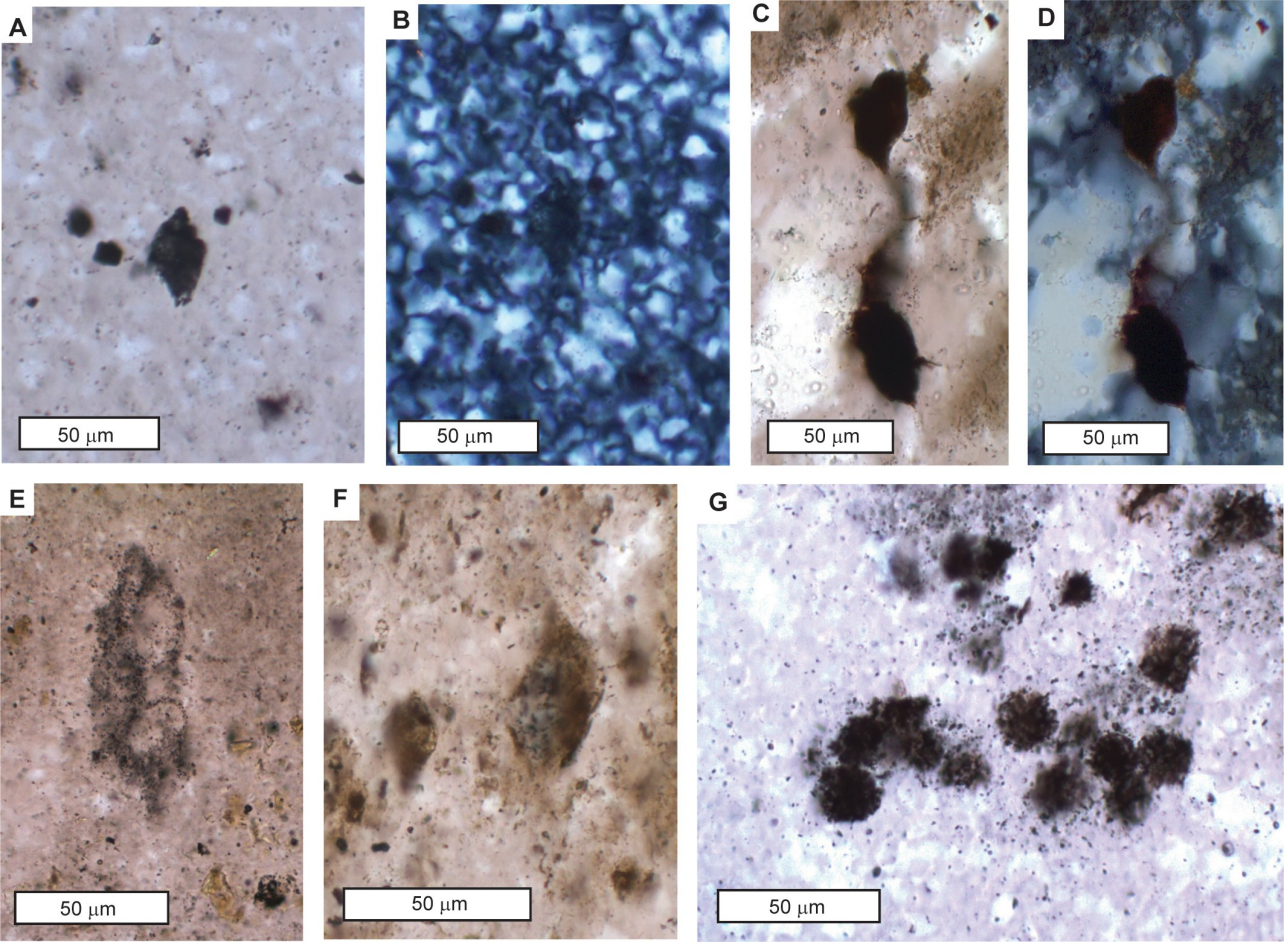
Microfossils from the Strelley Pool Formation near Marble Bar, with most views in plane polarized light, but some views in crossed nicols (**B, D**). Most (**A-F**) are enigmatic spindles cf. *Eopoikilofusa* [212], but spheroids are comparable with *Archaeosphaeroides pilbarensis* [198]. These thin sections are interpreted as A horizon of Wanta loam Paleosol (**A, B, G**), of Jurl clay loam paleosol (**F**), and of Ngumpu loam paleosol (**E**) and are archived in the Museum of Natural and Cultural History of the University of Oregon as R3795 (A, B), R3803 (C-D,F), R3807 (E), R3794 (G).

A better case can be made for early diagenetic Archean silicification [78], which is required to preserve organic microfossils in both the Apex chert (Fig 14 [10, 11]) and Strelley Pool Formation (Fig 15 [21–25]). Clasts of white chert of the Strelley Pool Formation and black chert of the Apex basalt are both found in pyroclastic breccia overlying the Strelley Pool Formation near Marble Bar, so predate eruption of Euro Basalt [9]. Microfossils would not remain had silicification not preserved them from decay during deposition. Early silicification also is indicated by redeposited silicified clasts in the Apex Chert [10, 89], which we confirm here (Fig 14A). Previously reported Strelley Pool Formation microfossils include robust large spindles, which have been accepted as genuine microfossils for reasons of carbon isotope composition, Raman spectroscopy, and three-dimensional imaging [8, 21–25, 90, 91]. However, the Apex chert microfossils have been questioned [6–8, 45] as disorganized skeins of abiotically produced organic matter structured by neomorphic crystal growth or accidental folding in a hydrothermal vent. Remapping of the locality here demonstrates instead a sedimentary chert bed, within a sequence of pillow basalts (Fig 6). Furthermore, subsequent research [5, 11, 91–93] supports the conclusion that Apex chert has some genuine microfossils with Raman spectroscopy, carbon isotopic analyses and three dimensional imaging techniques. Nuclear magnetic resonance and pyrolysis is also evidence for aromatic hydrocarbons of biological orgin [94], and ultrastructural examination also reveals organic microstructure [12]. Brasier et al. [6–8, 45] are correct in documenting decay, tearing, and folding of organic matter in the Apex chert, and such signs of decay are one of the criteria for distinguishing genune organic microfossils from mineral pseudomorphs [95]. Our own studies confirm microfossil deformation by neomorphic crystal growth (Fig 14B), common for Precambrian microfossils [96]. Back-scatter scanning electron micrographs of Apex chert show widespread exclusion of organic matter to interstices of neomorphic grains 5 μm in diameter. However, we also found a few microfossils straddling neomorphic crystal domains, and these are original organic structures (arrows in Fig 14B), unlike organic matter displaced by crystal growth. A comparable phenomenon can be seen in the Strelley Pool Formation, where spheroidal fossils have walls disrupted by smaller (2–3 μm) neomorphic quartz (Fig 15G), and so appear to have been intact [8, 25].

**Fig 16:**
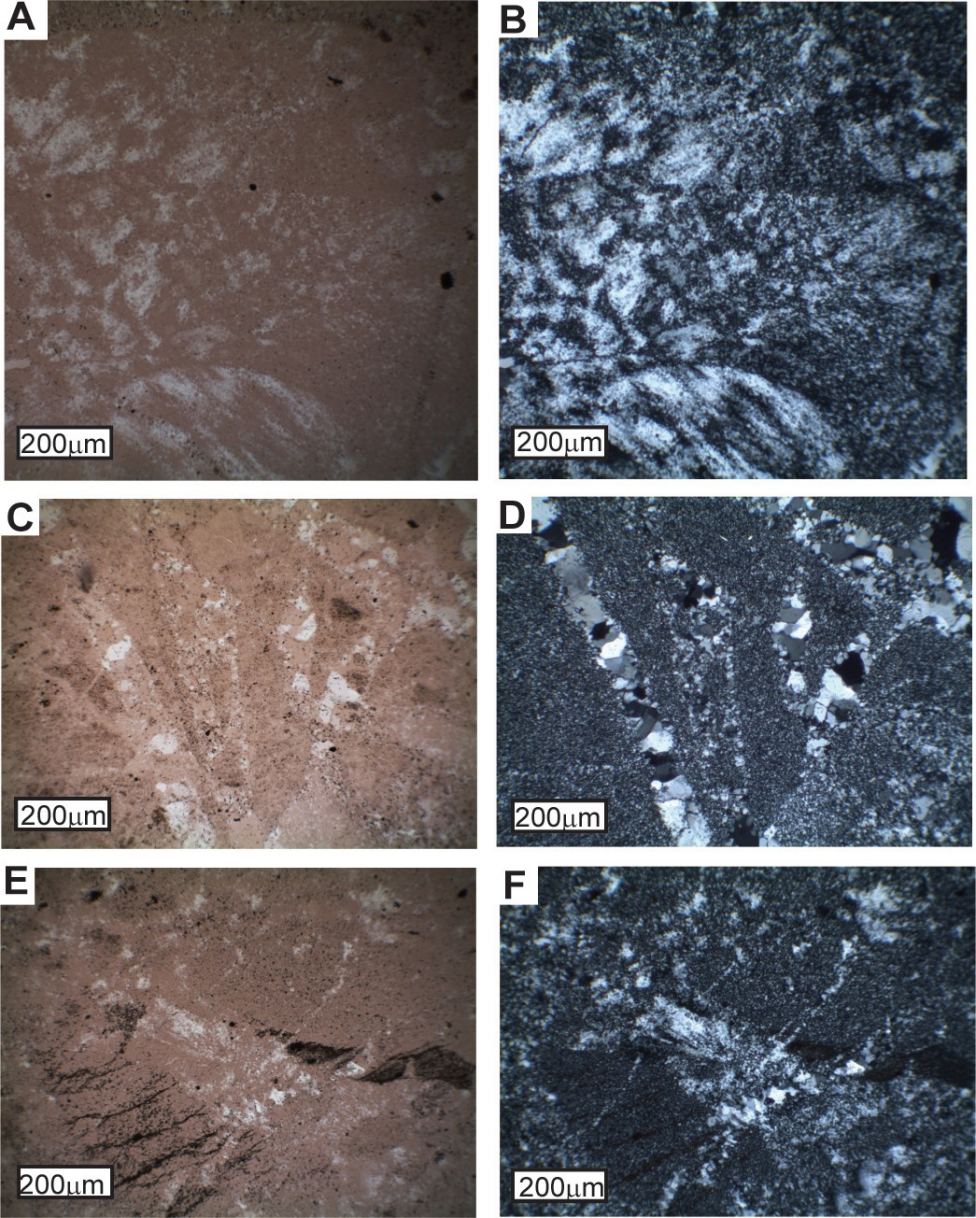
Evaporite sand crystal pseudomorphs in plan polarized light (left hand side) and under crossed nicols: **A, B**, nodularized botryoidal barite; **C, D**, nahcolite needles; **E, F**, searlesite (?) spherulite. Samples in Museum of Natural and Cultural History of the University of Oregon are (A-D) R34796 from By horizon of Wanta loam paleosol, and (E-F) R3800 from By horizon of Wanta silty clay loam paleosol.

Rare earth element analyses are evidence that replacive silicification of dolomite and siltstone of the Strelley Pool Formation was in surface or groundwater [50, 63], as envisaged for other Precambrian stromatolitic cherts [97, 98]. One source of silica is diagenetic alteration by dissolution and replacement within the zone of brine and fresh water mixing responsible also for dolomitization in a Precambrian world of higher marine silica concentrations than today before evolution of abundant siliceous plankton such as diatoms and radiolaria [97, 98]. A second idea is diagenetic remobilization of silica spicules secreted by sulphate-reducing bacteria [99, 100], which can be inferred from previous studies of microfossils and sulfur isotopes of the Strelley Pool Formation [23, 24, 101]. Silicification of evaporite minerals in the Strelley Pool Formation and Apex Chert is near total (Fig 16), whereas stromatolitic dolostones are only partly replaced, and this is evidence for a third alternative of formation as playa cherts, comparable with magadiite [102, 103]. Minerals such as barite (BaSO_4_), gypsum (CaSO_4_.2H_2_O) and nahcolite (NaHCO_3_) are precipitated at very high pH (>9), which mobilizes silica. Highly alkaline solutions dissolve opaline biogenic or volcanic silica in preference to quartz, forming silica gels in playas and intertidal flats [103, 104]. A model of caustic rather than neutral dissolution, perhaps aided by sulphate-reducing bacteria is appealing for silica permineralization of microfossiliferous Precambrian intertidal to supratidal cherts [98].

A final source of silica for basal quarzites, thin veins, and breccias of the Strelley Pool Formation is dissolved silica released by illitization of clays, and by pressure solution of sand grains [105]. Such silica is responsible also for late diagenetic neomorphic recrystallization, best seen under crossed nicols (Fig 15B and 15D), or back-scatter scanning electron microscopy (Fig 14B and 14D). Late diagenetic remobilization of silica is also revealed by geochemical evidence for illitization (Fig 11) and crushing of grain boundaries in thin sections of the Strelley Pool Formation (Fig 7A–7C).

### Are there paleosols below and within Strelley Pool Formation?

Paleosols within and below the Strelley Pool Formation have been controversial, denied by some [2, 3, 5–9], but supported by others [1, 4]. The following paragraphs address the simple question “Was it a paleosol?” A paleoenvironmental narrative on the significance of paleosols for Archean life and paleoenviromnents is deferred until next section.

### Field observations of paleosols

The geographically extensive pre-Strelley paleosol [1] proved to be very similar at every locality examined (Fig 3), including the new locality near Marble Bar (Figs 5B and 6). It is more than 20% sericitized clay to a depth of about 3 m, then declining amounts of sericite to none beyond 4 meters depth (Figs 3 and 13). It is conspicuous in the field: green-gray when fresh, but weathering bright orange in outcrop unlike surrounding red soil (Fig 5A and 5B). Clay of the profile formed at the expense of basaltic or other igneous rock fragments and corestones as expected for hydrolytic weathering. Furthermore, fragments of surface horizon clay are found as clasts in basal sandstones of the Strelley Pool Formation [18, 19], as evidence that weathering predated deposition of the overlying sequence. Highly birefringent pyrophyllite is also found in varying amounts between profiles, but little changed in abundance within and below individual profiles (Fig 13), so was a part of hydrothermally altered parent material [3]. The lower meter of these paleosols has corestones of less weathered parent material some 20–30 cm in diameter (Fig 5A). The upper meter has fine veinlets of contorted iron-manganese: in soil terminology these are mangans defining coarse blocky angular peds. This widespread thick paleosol is here given the non-genetic name Jurta pedotype, from a Nyamal language word for big [106]. These thick paleosols are comparable in clayeyness and weathering trends with the 3.0 Ga Jerico Dam pedotype of South Africa [42], but developed on pyrophyllitic-metabasalt rather than granite.

In addition to these thick clayey unconformity paleosols, thin alluvial paleosols were also recognized within the Strelley Pool Formation. These have been hiding in plain sight as cherty layers with pseudomorphs of evaporites interpreted [51] to “represent sedimentation in marginal hypersaline salinasnas and low energy coastal lagoons.” Sabkha, playa and salina facies are sedimentary facies but also soils classified as Gypsids [107], Solonchaks [108], or Sodosols [109]. Playas and salinasnas are inundated rarely, and their development of desiccation cracks, salt crystals, and shear planes are soil-forming rather than sedimentary processes [110]. The alluvial paleosols are in facies with trough cross bedding (Fig 5C) and ripple marks at stratigraphic levels previously interpreted as estuarine to littoral talus [51], stratigraphically below stromatolitic cherts and dolostones (Fig 3 [9, 18, 19]).

Paleosols were recognized in the field as massive chert beds with disrupted surface (A horizon) abruptly overlain by bedded sandstone, and grading down into nodularized or pseudomorphed barite (By horizon). The baritic pseudomorphs have grain inclusions and are also overgrown into nodular forms (Figs 5D and 16A and 16B) characteristic of “desert roses” of modern aridland soils [111], and of alluvial paleosols widespread in the 3.5 Ga Panorama Formation of Western Australia [27], 3.2 Ga Moodies Group of South Africa [112], and 3.0 Ga Farrel Quartzite of Western Australia [26]. An array of different kinds of alluvial paleosols were recognized and sampled (Fig 12) showing incipient bedding disruption (Ngumpu), small barite (Jurnpa), dense barite (Jurl), and with hematite-varved surfaces (Wanta), comparable with the array of Entisols to Aridosols recognized in Proterozoic quartz-rich alluvial sequences, but mainly green-gray rather than red [27].

### Laboratory tests for paleosols

Weathering trends in rocks unconformably below the Strelley Pool Formation are duplicated in paleosols discovered within the Strelley Pool Formation, so present a consistent view of distinctive Archean styles of soil formation. A first laboratory test for paleosols is petrographic point-counting, which assumed 5 μm neomorphic recrystallization of clay and pervasive early diagenetic silicification, as documented by transmission electron microscopy [15]. The thin alluvial paleosols are comparable with the thick unconformity paleosols in showing destruction of rock-fragment grains upwards in the profile coordinated with increases in clay to a degree proportional to thickness and degree of physical alteration of the profiles (Figs 12 and 13). Weakly developed profiles with relict bedding such as Jurnpa and Ngumpu show muted mineral differentiation compared with well developed Jurl, Wanta and Jurta profiles. These are trends documented in many Precambrian paleosols [113].

A second test for paleosols is geochemical: molecular weathering and concentration ratios of major oxides (Figs 12 and 13). Geochemical depth functions are muted, with expected elemental mobilization related to proportional enrichment of clay or of evaporite sand crystals and nodules. Iron was lost from all except Wanta profiles, which have surface hematite lamination. Thick Jurta profiles show subsurface loss of lime and magnesia and soda, in contrast to nodular intra-Strelley profiles with gains of these elements in proportion to nodule abundance. Elemental losses vary from large in Jurta, Jurl and Wanta profiles, but are muted in Jurnpa and Ngumpu profiles. This variation in weathering inversely proportional to preservation of bedding, is also very like soils and paleosols [40]. Soda was close to limits of detection in all samples, but like potash, sometimes enriched, perhaps due to illitization (Fig 11) or to acid-sulphate weathering [114].

**Fig 17:**
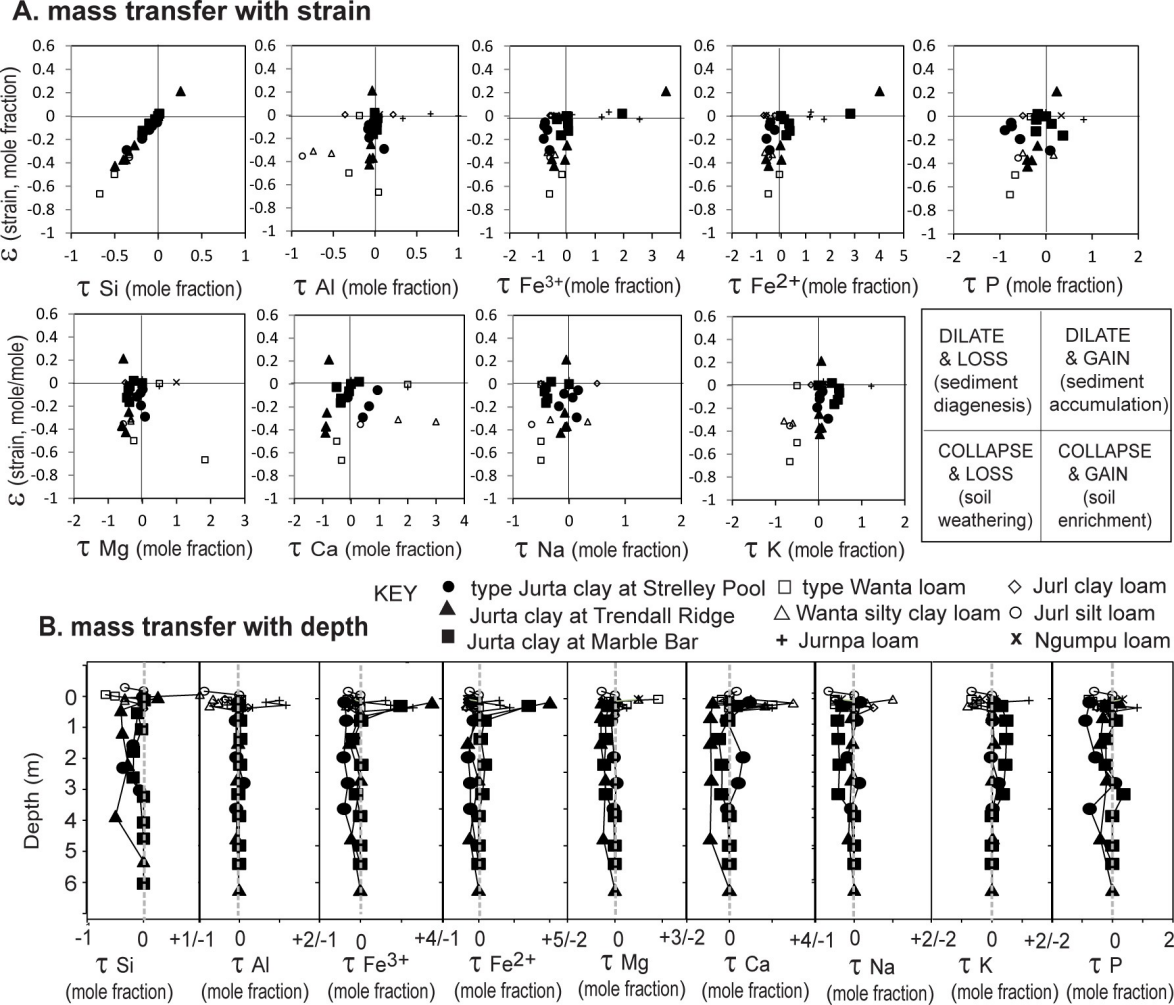
Tau analysis of paleosols within and below the Strelley Pool Formation. Individual paleosols vary in degree of weathering, but are mainly in the collapse-and-loss field of soils, rather than the dilate-and-gain field of sediments [41].

A third test for paleosols is chemical mass balance or “tau analysis”, which normalizes to a stable constituent in parent material (titania used here), and calculates mole fraction losses and strain from weight percent and bulk density of each sample [41]. Both the unconformity profiles (Jurta) and alluvial profiles (Jurl and Wanta) show mostly within-bed changes in the collapse-and-loss quadrant of soils rather than the dilate-and-gain quadrant of sediments (Fig 17). Surface horizons of some Jurta paleosols, and of weakly developed Ngumpu and Jurnpa paleosols show element gain but modest dilation of aeolian-fluvial additions incompletely digested by pedogenesis. Hydrothermal alteration documented for Jurta profiles underlying the Strelley Pool Formation [3] does not compromise tau analysis, because pyrophyllitic alteration of basalts predated soil formation and is the assumed parent material (Fig 13). Bed-scale soil-like strain-transfer patterns have persisted despite later regional greenschist facies metamorphism of the pre-Strelley rocks [1] and syn-sedimentary silicification of the Strelley Pool Formation [19]. Tau analysis reveals that regional metamorphism did not destroy or homogenize the original signal of within-bed pedogenesis.

**Fig 18:**
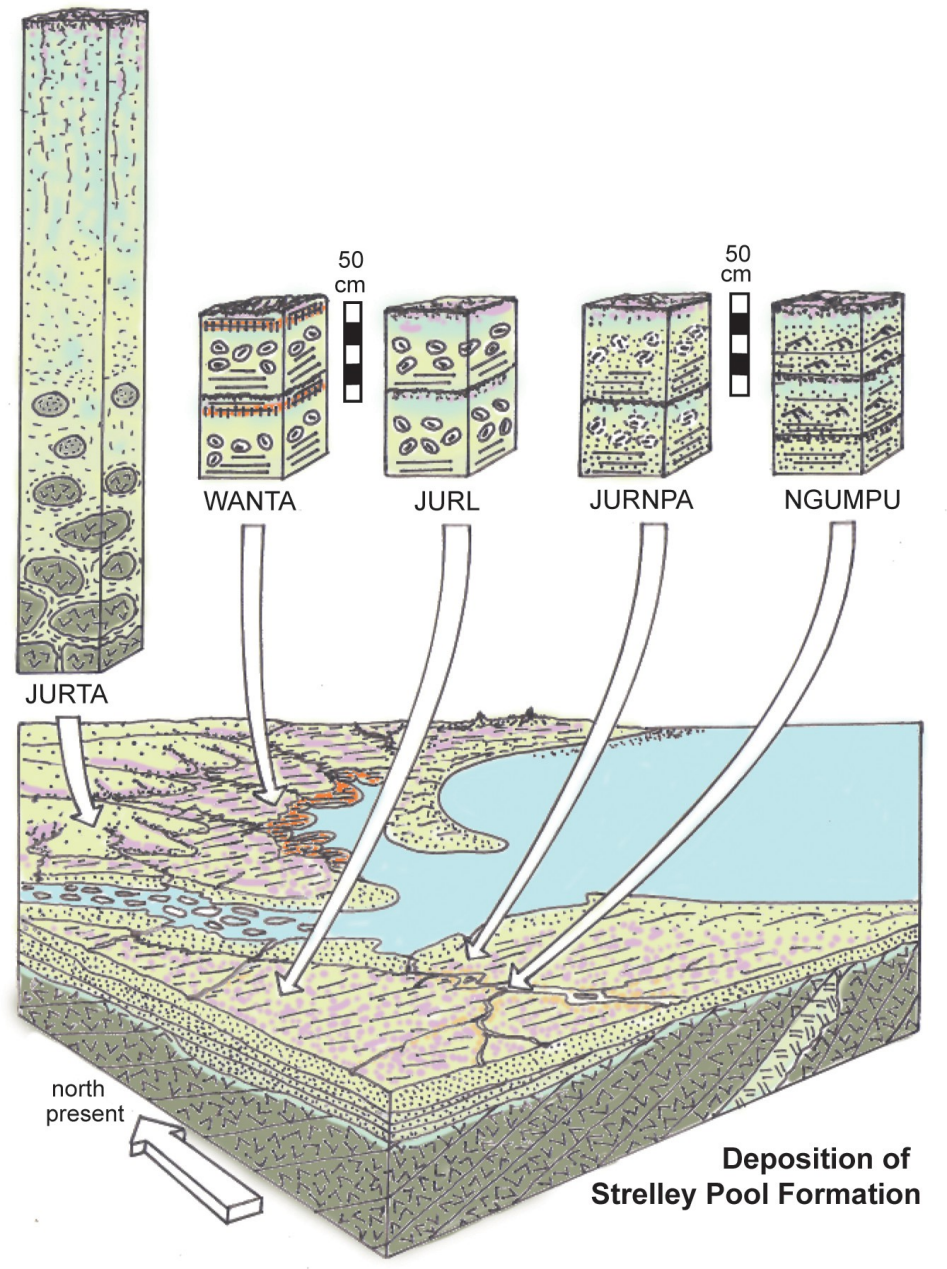
Graphical abstract of reconstructed paleosols below and within the Strelley Pool Formation.

Each kind of paleosol (Table 2) within and below the Strelley Pool Formation offers a variety of paleoenvironmental information independent of sedimentological and paleogeographic studies (Table 3), and these are justified in the following paragraphs to generate a model for these ancient landscapes and their soils (Fig 18).

**Table 2:** Paleosol definition and classification within and below Strelley Pool Formation.

Pedotype	Nyamal (*52*)	Diagnosis	US taxo-nomy [107]	FAO map unit [108]	Australian Classificat-ion [109]
Jurnpa	Cold ashes	Black massive cherty surface (A horizon) over barite mottle pseudomorphs in trough cross-bedded sandstone (By horizon)	Ustept	Eutric Cambisol	Gray-orthic Tenosol
Jurl	Salt	Black massive cherty surface (A horizon) over barite crystal pseudomorphs in gray chert (By horizon)	Cambid	Orthic Solonchak	Gray Sodosol
Jurta	Big	Cracked surface (A horizon) over thick green sericite (Bw horizon) and sericite with corestones (C horizon)	Udept	Dystric Cambisol	Gray Kandosol
Ngumpu	Narrow	Black massive cherty surface (A horizon) over laminated gray chert (C horizon).	Fluvent	Eutric Fluvisol	Stratic Rudosol
Wanta	Crazy	Red cracked and laminated surface (A horizon) over barite mottle pseudomorphs (By hoprison)	Halaquept	Gleyic Solonchak	Gray-orthic Tenosol

**Table 3:** Paleosol interpretations within and below Strelley Pool Formation.

Pedotype	Climate	Organisms	Topography	Parent material	Soil duration
Jurnpa	Humid (1037 ± 182 mm MAP) temperate (6.9 ± 0.2°C MAT)	Microbial earth dominated by methanogens and actinobacteria, with purple sulfur bacteria	Streamside levee	Quartz-lithic sand	1,000–69,000 years
Jurl	Humid (1405 ± 182 mm MAP) temperate (10.6 ± 0.2°C MAT)	Microbial earth dominated by purple sulfur bacteria, with actinobacteria and methanogens	Floodplain	Quartz-lithic silt	2,000–82,000 years
Jurta	Humid (1376 ± 182 mm MAP) temperate (10.6 ± 0.2°C MAT)	Microbial earth	Low hills	Basalt	100,000–300,000 years
Ngumpu	Not diagnostic for climate	Microbial earth dominated by methanogens and actinobacteria, with purple sulfur bacteria	Streamside bar	Quartz-lithic sand	10–1000 years
Wanta	Not diagnostic for climate	Microbial earth dominated by iron-oxidizing bacteria	Floodplain swale	Quartz-lithic sand	2,000–82,000 years

Note: MAP is mean annual precipitation and MAT is mean annual temperature.

## Discussion: Interpreting paleoenvironments of Archean paleosols

The following paragraphs offer a multifaceted interpretive narrative of what paleosols in and below the 3.3 Ga Strelley Pool Formation can potentially reveal about Archean terrestrial weathering and life.

### Topographic setting

The sedimentary setting of the Strelley Pool Formation was coastal: alluvial levees, floodplains, estuaries, littoral talus and stromatolitic intertidal flats [8, 18–20, 46, 51]. Erosion resistant lithologies, such as chert, in underlying rocks created local ribs with paleorelief of up to 3 m (Fig 5A and 5B), but the wide extent of only 30-m-thickness of Strelley Pool Formation over some 30,000 km^2^ (Fig 1), is evidence of regional low relief. One exception is a likely paleocanyon 1 km deep near Doolena Gap, on the highway north of Marble Bar (Fig 1), where 1000 m of Strelley Pool sandstone has eroded all the way through Apex Basalt and Marble Bar Chert into the Duffer Formation [53]. The Strelley Pool Formation thins to the west of Strelley Pool [46, 51] and in that direction also the dip of the angular unconformity is greater than dips to the east (Fig 4). Those relationships and the paleocanyon at Doolena Gap [53] are evidence of paleoslope down to what is now southeast. This Pilbara Craton of tonalite-trondjheimite-granodiorites of the 3500–3460 Ma Callina Supersuite to the west, and the 3450–3420 Ma Tambina Supersuite to the north, predated large granitic complexes of the 3325–3290 Ma Emu Pool and 3275–3225 Ma Cleland Supersuites [115] and domal tectonics [116]. The land mass during deposition of the Strelley Pool Formation was about 540 km in diameter before a 3200 Ma rifting event that separated Karratha and Kurrana terranes [117].

Jurta paleosols of the pre-Strelley landscape show clay formation and corestones to a depth of 4 m, which after decompaction (following equation in Table 1) was 9 m, and as in modern soils, this may represent a minimum depth of the water table [118, 119]. The pre-Strelley paleosol has been considered as deep as 50 m [1], but our examination of outcrops near Strelley Pool (Fig 2A) suggests that this was not weathering but deep hydrothermal pyrophyllitic alteration [3]. The pyrophyllite alteration cupola to the North Pole Monzogranite was also mistaken as a paleosol 35 m thick [4], based on misidentification of red beds some 12–20 m below the surface as Archean laterites (Fig 2B). Our examination of these ferruginous bands found that they were pods of banded iron formation within the Panorama Formation, rather than nodular, pisolitic or massive laterite [36].

Paleosols within the lower Strelley Pool Formation formed on flat coastal depositional landscapes of estuaries, rivers and floodplains [18, 19, 46]. Permanent water table in this floodplain was at least 25 cm below the surface of the paleosols. Above that level, evaporite sand crystals and nodules demonstrate replacive growth characteristic of dry soils [40], evident from sedimentary inclusions and dusty rims (Fig 16). Such soil crystals are distinct from crystals precipitated from open water or saturated sediments that are clean of inclusions because excess water allows growth by displacement. Clean evaporite crystals are well known from marine evaporites [120, 121], playa lakes [102, 103], and spring tufas [122–124]. Sulfur-spring barites and other crystals are aligned with original bedding [122–124], rather than randomly oriented as in the Strelley Pool Formation (Fig 16).

### Parent material

Pre-Strelley Jurta paleosols developed on a bedrock landscape of varied formations (Fig 4): Double Bar Basalt and Coucal Formation at Strelley Pool, Mt Ada Basalt at Steer Ridge, Panorama Formation at Trendall Ridge, and Apex Basalt near Marble Bar [7, 28, 125 126]. Parent material variation can be seen on a local scale near Marble Bar, where the Apex Basalt is deeply and recessively weathered, but cherty interbeds were little weathered, and formed topographic ribs on the ancient landscape (Fig 5B).

Unlike pre-Strelley Jurta paleosols (Fig 13), those within the Strelley Pool Formation (Fig 12) formed on sediments eroded from a prior cycle of weathering and do not present materials identifiable as unweathered in petrographic thin sections. Paleosols within the Strelley Pool Formation are developed on quartzofeldspathic sand with few clay and rock fragments, including variably weathered volcanics, pyrophyllitic schist, and both recycled and intraformational chert. No dolostone was detected within these paleosols or their parent material, but dolostone is found in stromatolitic parts of the formation, and could have been recycled into paleosols. Silicification of much of the stromatolitic part of the Strelley Pool Formation has converted these beds to chert [18, 19], and carbonate clasts in the paleosols would have been rendered indistinguishable from other rock fragments by silicification.

### Time for formation

At a reconstructed 9 m thick and lacking duricrusts, the pre-Strelley or Jurta paleosols (Fig 9) are strongly developed, rather than very strongly developed in a field scale devised for modern soils [40]. They endured a long time of soil formation to convert a variety of hydrothermally altered basalts and tuffs (Fig 7E and 7G and 7I) to sericitized clay (Fig 7D and 7F and 7H). Taxonomic considerations illustrate differences with modern soils that compromise this interpretation. Such well developed soils today have argillic horizons of Ultisols or Alfisols, or thick kaolinitic horizons of Oxisols, but Jurta Paleosols have insufficient subsurface clay enrichment for an argillic horizon, and are not chemically weathered enough for an oxic horizon (Fig 13). Thus Jurta paleosols present a sequence of horizons (A-Bw-C) of Inceptisols, which are generally weakly developed [40]. Their combination of well drained but chemically reduced is also not a combination found today [117, 118], and a new soil order of Viridisols (green clays) has been suggested to accommodate them [127]. Viridisol has not been formally ratified [107], and for the moment these paleosols are placed in the order Inceptisol.

Paleosols within the Strelley Pool Formation show varying degrees of destruction of bedding and ripple marks which indicate moderate (Jurl, Wanta) to weak (Jurnpa) and very weak development (Ngumpu) in a field scale for soils [40]. Once again there are confounding differences, because moderately developed soils and paleosols are marked by nodules of gypsum [128], or low-magnesium calcite [129], but nodules and crystals in paleosols of the Strelley Pool Formation appear to be barite and nahcolite for which no modern chronosequences have been studied, and which now need special hydrological and microbial constraints [114, 130].

Archean paleosols formed under very different conditions than modern soils, but such tenuous comparisons are needed to estimate durations of soil formation. Durations of post-Devonian forested paleosols on bedrock can be estimated from a variety of chronofunctions (Table 1) giving several orders of magnitude spread of durations (S8 Table). Of the various options of largely temperate humid chronofunctions the extraordinarily high rate of regolith production inferred [131] from uranium dating of weathering of pyritic black shales in Pennsylvania is preferred here: 138,724 ± 24,069 years for the Jurta clay at Strelley Pool, 199,416 ± 20,069 for the Jurta clay at Trendall Ridge and 95,373 ± 24,609 for the Jurta silty clay loam at Marble Bar. Longer estimates for soil formation come from other chronofunctions [132–134]. Pyritic black shales show acid-sulphate weathering [135], which is more aggressive than carbonic acid weathering. For example, acid sulphate weathering of baritic and gypsiferous upland soils of the Lufkin Series in Texas reached 220 cm thickness in only 15,155 years [114]. With high CO_2_ comes acid rain (pH 4.0–4.5) and biogenic sulfuric acid could further acidify soil water (pH 2–3 [4]), and increase soil production rates. Acid sulphate weathering of Jurta paleosols is compatible with their pyrite content, and the abundance of sulphate and likely sulfur bacteria in the overlying Strelley Pool Formation [23, 24]. Archean bedrock paleosols were not necessarily thin, nor poorly differentiated chemically, as has long been apparent from the study of Archean paleosols at major geological unconformities [42, 113, 136, 137].

Moderately developed alluvial paleosols of forested post-Devonian paleosols have discrete nodules or sand crystals, which now take millennia to coalesce [40]. Two transfer functions for estimating paleosol ages (Table 1) have been derived from diameter of low-magnesium calcite nodules in radiocarbon-dated soils of New Mexico [129], and abundance of gypsum crystals of the Negev and Atacama Deserts [128]. If the sand crystals and nodules of the Strelley Pool Formation were calcite and had diameters measured in the field, the duration of 18 Jurl paleosols average 3,843 ± 1,800 years, 3 Jurnpa paleosols average 4,283 ± 1,800 years, and 8 Wanta paleosols average 3,568 ± 1,800 years (S9 Table). If on the other hand, they were gypsum crystals and had densities measured in the field, the duration of 18 Jurl Paleosols average 42,322 ± 15,000 years, 3 Jurnpa paleosols average 31,025 ± 15,000 years, and 8 Wanta paleosols average 40,162 ± 15,000 years. These estimates are orders of magnitude different, but provide a generous envelope of possibilities. They are not strictly relevant because pedogenic nodules and crystals in the Strelley Pool Formation are silica pseudomorphs, probably mainly barite and nahcolite [37], for which no modern chronofunctions are available. There is no prospect for obtaining a chronofunction for precipitation of either mineral from Holocene soils, because nahcolite requires higher than modern CO_2_ partial pressures [130], and barite nodules require anoxic sulfur bacterial biomineralizers [114]. Paleosols with these minerals are still few and poorly known [114, 138]. Although accurate estimates for duration of soil formation in the Archean remain elusive, many millenia were needed for Jurl and Wanta and proportionally less for Jurnpa and Ngumpu Paleosols. Archean alluvial paleosols were not necessarily poorly developed or unrecognizable.

### Paleoclimate

Chemical Index of Alteration for paleosols both below (Fig 13) and within the Strelley Pool Formation (Fig 12) reveals temperate weathering, neither frigid-glacial nor tropical [43]. Application of more specific pedogenic paleothermometers to Archean paleosols is risky, because most are based on soils of modern woody vegetation [139, 140]. The best choice for Archean paleosols predating the evolution of modern vegetation is a palaeothermometer (Table 1) based on modern soils under tundra vegetation of Iceland [141], although even these lichens and shrubs are very different from microbial communities responsible for weathering of Archean paleosols [142]. These calculations give temperate mean annual paleotemperatures for the lower A horizons of Jurl (10.6 ± 0.4°C) and Jurnpa (6.9 ± 0.4°C) paleosols of the Strelley Pool Formation. The alkali index paleotheromometer [139] gives comparable results of 11.6 ± 4.4°C for Jurl, and 12.6 ± 4.4°C for Jurnpa, but paleosol weathering index [140] was much warmer with 33.1 ± 2.1°C for Jurl, and for 37.9 ± 2.1°C Jurnpa. The pre-Strelley Jurta paleosols all gave similar results for the Iceland paleothermometer [141]: 11.8 ± 0.4°C at Strelley Pool, 11.5 ± 0.4°C at Trendall Ridge and 10.5 ± 0.4°C near Marble Bar. Applying the alkali index paleothermometer [139] gave 11.0 ± 0.4°C at Strelley Pool, 11.6 ± 0.4°C at Trendall Ridge and 10.6 ± 0.4°C near Marble Bar, but the paleosol weathering index [140] was again much warmer, 25.8 ± 0.4°C at Strelley Pool, 25.8 ± 0.4°C at Trendall Ridge and 25.2 ± 0.4°C near Marble Bar.

Similar “temperate paleoclimate” (<40°C) has been inferred for the 3.4 Ga Buck Reef Chert of South Africa, based on evidence of oxygen and hydrogen isotopic composition [143]. Earlier unrealistic estimates of 55–85°C come from oxygen isotopic evidence alone [144]. Substantial (6–38 volume %) original quartz in the Strelley Pool Formation is evidence of temperatures less than 75°C (Figs 12 and 13), because quartz dissolves at higher temperatures [145]. This line of inference may not be appropriate for little-weathered sediment of tectonically active terranes [146], but facies analysis and grain size of the Strelley Pool Formation are evidence of subdued tectonic and volcanic activity of a continent-sized landmass [46]. Temperate paleotemperatures are consistent with mid-paleolatitude position of the Strelley Pool Formation: between 20.5 ± 5^o^ for the ca. 3460 Ma upper Apex Chert [147], and 59.0 ± 8.8^o^ for the 2.86 ± 0.2 Ga Millindinna Complex of the West Pilbara [148].

A widely used paleohyetometer [139] based on temperate soils of North America uses chemical index of alteration without potash (CIA-K of Table 1). This paleohyetometer gives humid mean annual precipitation for lower A horizons of Jurl (1405 ± 182 mm) and Jurnpa (1037± 182 mm) paleosols, as well as Jurta Paleosols of Strelley Pool (1552 ± 182 mm), Trendall Ridge (1077 ± 182 mm) and Marble Bar (1376 ± 182 mm). These data support other indications of deep weathering such as light rare earth enrichment of some parts of the Strelley Pool Formation [50]. However, other parts of the formation have flat or light rare earth depleted compositions betraying marine influence [63, 79].

Jurta paleosols beneath the Strelley Pool Formation are clayey and lack salts as expected in humid climate, but abundant evaporite pseudomorphs in paleosols within the Strelley Pool Formation are suggestive of arid paleoclimates [128]. Paleoclimatic implications depend on the kinds of minerals, but Archean evaporite minerals have been controversial because they are mainly preserved as silica pseudomorphs (Fig 16). Thus their original nature can rarely be determined by chemical analysis, and depends on crystallographic appraisal of interfacial angles, already reported for the 3.0 Ga Farrel Quartzite [37], 3.2 Ga Moodies Group [112], 3.4 Ga Kromberg Formation [149], 3.3 Ga Strelley Pool Formation [18, 19, 48, 51, 150], 3.5 Ga Apex Chert [6, 38], and 3.5 Ga Dresser Formation [151, 152]. Minerals inferred from pseudomorphs include barite (BaSO_4_), selenite-gypsum (CaSO_4_.2H_2_O), aragonite (CaCO_3_), and nahcolite (NaHCO_3_), but the only evaporite mineral still detectable as chemical remnants is barite [6, 37, 151, 153, 154]. Counting against gypsum for the Apex and Strelley Pool Formations are the lack of selenite fishtail twinning [152], and apparent absence of equant-orthorhombic anhydrite (CaSO_4_), which forms from elongate-monoclinic gypsum during shallow burial [155]. Some supporting evidence for nahcolite may come from microscopic spherulites with very strong cleavage (Fig 16E and 16F), comparable with the phyllosilicate searlesite (NaBSi_2_O_5_(OH)_2_)), a common associate of nahcolite in soda lakes [156]. Crystal fans of nahcolite and aragonite have been confused in the past, but can be distinguished by hexagonal and square cross sections respectively [149]. Our study did not encounter local pods of abundant needle-like crystals interpreted as as gypsum or aragonite [28, 51], and as aragonite [20, 46], and found only a few thin sections with hexagonal needles like nahcolite (Fig 16C and 16D).

Archean silicified barite and nahcolite crystals have been interpreted as evidence of hydrothermal veins [38], or evaporating saline water [18, 37, 51, 149–151, 154]. These alternatives may be reasonable for the Apex chert which shows evidence of cleanly corroded crystal moulds (Fig 14C and 14D), remnant clear crystals of barite [6], and other evidence for burial alteration [38, 47]. In contrast, the pre-Strelley paleosol and the lower Strelley Pool Formation lacks the Raman spectroscopic [65], rare earth element [63], or distinctive mineral signatures of hydrothermal alteration [2]. Nor do the crystal casts form discrete beds of interlocking mosaics or inclusion-free crystals of evaporites precipitated from a water column [121]. Instead, the crystals are scattered within discrete horizons a set distance below (Fig 5D), but not at the tops, of the beds, like evaporites in soils [127]. Furthermore, crystal pseudomorphs in the Strelley Pool Formation show common sedimentary inclusions, nodularized overgrowths of crystal terminations, and lack of disruption of bedding planes (Fig 16). Nahcolite and barite are widespread as crystals, spherulites and nodules in sodic soils [157, 158] and playa lake crusts [102]. Nahcolite can form from the weathering of natrocarbonatite lavas [159], but carbonatite parent tuffs have distinctive enriched light rare earths [138], unlike flat rare earth patterns of the Apex Chert and Strelley Pool Formation [47, 63, 79]. Within playa lakes of semi-arid regions nahcolite is precipitated within sediment below the surface [102, 103], as also appears true of pre-Quaternary evaporites [104, 156, 160]. Modern pedogenic nahcolite and barite crystals and spherulites are mostly microscopic [157, 158], but some soils have barite nodules as much as 3.8 cm in diameter [114] like those of paleosols [114, 138]. Nahcolite has been reported in semiarid to arid soils and playa lakes, receiving 200–500 mm mean annual precipitation [157, 158], but is more strongly controlled by partial pressure of CO_2_ than by paleoclimate [130]. Pedogenic barite is known in humid forested soils with 1000–1200 mm mean annual precipitation, but under special circumstances, including microbially induced precipitation, or redox changes at the water table, unrelated to paleoclimate [114, 138]. Barite is favoured by acid sulphate weathering at low pH (<3), but gypsum at higher pH (4–9 [135]). There is as yet no clear relationship between depth of barite or nahcolite and mean annual precipitation as there is for gypsum [128]. Nevertheless, individual beds of the Strelley Pool Formation do show nahcolite and barite confined to a specific horizon (By) within the bed, and variably nodularized as in gypsic horizons of paleosols of Earth [128] and Mars [110, 161]. Salts may have been suppressed from thin (5–10 cm) uppermost horizons of Archean and Martian paleosols by surficial moisture or carbonic acid from biological productivity, as in desert soils of Earth [162].

### Ancient atmospheric CO_2_

Levels of soil CO_2_ can be approximated using a silicate weathering paleobarometer, which has been widely applied to rocks altered as much as greenschist facies [163], because higher temperature pyrophyllitic alteration did not alter the paleosols, but created a parent material below the angular unconformity [1, 4]. Jurta paleosols are especially suited for this because they deeply weathered profiles with clear losses of alkali and alkaline earth cations, unlike alluvial paleosols with salt pseudomorphs (Jurl, Jurnpa, and Wanta), or minimally developed paleosols (Ngumpu). Equations and variables for this CO_2_ paleobarometer are detailed in Table 1, and a full Gaussian error analysis [164] was conducted for this study (S10 and S11 Tables). This paleobarometer is inversely proportional to duration of soil formation [163], and a variety of durations were calculated (S8 Table). A maximal estimate of CO_2_ comes from modern acid sulphate weathering [131], but much lower estimates from other chronofunctions [132–134]. Maximal levels of atmospheric CO_2_ calculated by this means are 3170 ± 446 ppmv (11.3 ± 1.6 times preindustrial atmospheric level or PAL of 280 ppmv) for the Jurta paleosol at Strelley Pool, 2011 ± 237 ppmv (7.18 ± 0.8 PAL) for the Jurta paleosol at Trendall Ridge, and 3473 ±1 34 ppmv (8.8 ± 0.5 PAL) for the Jurta paleosol at Marble Bar. These are all above levels of 945 ppm soil CO_2_ at temperatures of 7–11°C required for precipitation and maintenance of nahcolite [130], a mineral inferred for the Wanta paleosol (Fig 16C and 16D). Nahcolite is rare in surficial environments today because unstable under current atmospheric levels of only 400 ppm CO_2_, and prone to recrystallizing to trona (Na_3_(CO_3_)(HCO_3_)·(2H_2_O) or natron (Na_2_CO_3_·10H_2_O). Archean soil-respiration CO_2_ levels are unknown, but could be determined if gypsic or calcic paleosols were found, and probably were 500–1000 ppmv (1.8–3.6 PAL) as in modern salty soils of deserts [162]. Atmospheric CO_2_ then would be the difference, some 1011–2670 ppmv (3.6–9.5 PAL), which is still above the nahcolite constraint [130].

Higher Archean CO_2_ levels (30,000 to 300,000 ppmv or 100–1000 PAL) have been inferred [165] from nahcolite formation at assumed Archean temperatures of 70°C, but such high temperatures are unlikely given oxygen and hydrogen isotopic studies [143], preservation of quartz [145], and application of paleosol paleothermometers used here [139–141]. Minimum levels of 2500 ppmv (8.9 PAL) CO_2_ have been inferred from weathering rinds on 3.2 Ga ferrous-carbonate clasts [166], but these fluvial pebbles may have been separated from the atmosphere by groundwater. Archean estimates of some 3,000 pmv (10.7 PAL) CO_2_ come from iron mineral stability in banded iron formations [165, 167], but these also reflect subaqueous rather than atmospheric levels. A maximum level of 36,000 ppmv (129 PAL) CO_2_ was inferred from paleosols ranging in age from 2.75–2.2 Ga because of their apparent lack of siderite [168]. This estimate suffers a variety of problems, such as choice of metamorphic rather than pedogenic mineral thermodynamic data [163]. An estimate of 1,500–9,000 ppmv (5–32 PAL) CO_2_ for 3.0 Ga comes from chemical weathering trends in the Jerico Dam paleosol of South Africa [42], and is comparable with estimates derived here [163]. This much CO_2_ would give acid rain (pH 4.0–4.5 [4]), and soil-microbial CO_2_ and H_2_SO_4_ could drop soil water pH to 3 favouring abundant barite precipitation observed [135].

Even the most extreme of these estimates is inadequate for a greenhouse capable of maintaining likely temperate Archean paleotemperatures [143] given the faint young sun. Other greenhouse gases are needed, including water vapour, CH_4_, C_2_H_6_, SO_2_ and OCS [169–171]. Much methane would have come from methanogenesis and would persist under low (160 ppm) H_2_ values likely for the Archean [171]. Three times the current mass of N_2_ and a H_2_ mixing ratio of 0.1 in the atmosphere, would also have created an adequate greenhouse [172]. However, N_2_ in the atmosphere was limited to 1.1 to 0.5 bars judging from nitrogen and argon isotopic ratios in fluid inclusions of the 3.5 Ga Dresser Formation of Western Australia [173]. The gas SO_2_ proposed theoretically for early Mars [174] and early Earth [175] is compatible with the abundance of sulphate in paleosols of the Strelley Pool Formation.

### Ancient atmospheric O_2_

Deeply weathered pre-Strelley Jurta paleosols can also be used to evaluate soil exposure to atmospheric O_2_, thus revising earlier estimates based on a very different concept of the pre-Strelley paleosol [4, 136]. This was done by modifying the silicate weathering paleobarometer [163], using integrated whole profile oxidation of iron instead of base loss (Table 1), the same estimates for duration of soil formation, and the following standards for O_2_ diffusion constant in air ($D_{O_{2}}\;=\;0.203\pm 0.024$ cm^2^/s [176]), ratio of diffusion in air/soil (α = 0.1 ± 0.02) and Henry’s Law constant for O_2_ ($K_{O_{2}}$ = 0.00125 ± 0.00005) [177]. Gaussian error has been calculated as for CO_2_ (S12 and S13 Tables). The results are 1788 ± 4006 ppm (0.008 ± 0.016 times preindustrial atmospheric level or PAL) for the Jurta paleosol at Strelley Pool, 1124 ± 5085 ppm (0.005 ± 0.024 PAL) for the Jurta paleosol at Trendall Ridge, and 2181 ± 3018 ppm (0.01 ± 0.014 PAL) for the Jurta paleosol at Marble Bar. The Marble Bar result is regarded as the most accurate because least compromised by burial illitization (Fig 11). These are maximal levels because based on the short soil formation times likely during acid sulphate weathering [131], rather than other modern chronofunctions [132–134]. Uncertainties are large and similar to the estimate, which should be taken as a rough approximation [27], though significantly lower O_2_ than a previous estimate from these paleosols [4].

Low levels of atmospheric oxygen are evident from paleosols within and below the Strelley Pool Formation because of their high ferrous to ferric iron ratios (Figs 12 and 13), comparable with swamp soils today exhausted of oxygen by microbial respiration [40]. However good drainage of the Strelley Pool Formation paleosols is indicated by dissolution casts and nodules of evaporitic mineral pseudomorphs (Fig 16). Laminations of hematite and goethite are distinguish the surface of Wanta paleosols (Fig 7J), but all other paleosols within the Strelley Pool Chert are gray green and unoxidized, including paleosols within and below the Strelley Pool Formation sampled by deep drilling (Coonterunah Core no. 8 [85]). The pre-Strelley paleosol in core and outcrop is gray and less than 4 m thick, and entirely postdates a thick pyrophyllitic hydrothermal alteration aureole [2, 3]. The pre-Strelley paleosol does not include laterite at Trandall Ridge [4], because these red beds are stringers of banded iron formation in parent material of Panorama Formation well below the paleosol. Munsell hue is a guide to Archean versus Phanerozoic iron oxides, because in most cases oxidation of the modern outcrop is goethite of brownish red color (Munsell 10YR-7.5YR), whereas Archean hematite is red (Munsell 5R to 10R), like a fire engine. There are some exceptions where Archean rocks are locally stained by overlying Mesozoic and Cenozoic paleosols [178, 179], or local oxidation along deep fissures [180, 181]. The 3.5 Ga Marble Bar Chert, an aquatic banded-iron formation, also shows original hematite, again as sampled by deep drilling [182]. Hematite banded iron formation and a pre-Strelley paleosol interpreted as lateritic have been used to argue for 100,000 ppm (0.48 PAL) atmospheric O_2_ back to 3.8 Ga [4]. However, hematite in banded iron formations formed by chemolithotrophy of iron-oxidizing bacteria under chemically reducing conditions [183, 184]. These observations support the idea that Archean redox distribution was upside-down compared with modern: oxidized biosynthetic hematite accumulated in stagnant waterlogged soils, lakes and oceans, but overlying water and air remained chemically reduced [185, 186]. Thus, the red surface of Wanta paleosols can be taken as evidence of an anaerobic microbial community adapted to waterlogging, whereas other communities of anaerobic but well drained soils maintained chemically reduced iron minerals. This redox distribution is upside down compared with today’s red and oxidized well drained soils, but gray-green and chemically reduced waterlogged soils [40].

Low oxygen calculated here from paleosols within and below the Strelley Pool Formaton is broadly similar to other Archean estimates of atmospheric composition from paleosols. Estimates of atmospheric O_2_ based on Jurta paleosols are a little higher than 20–1,000 ppm (0.00095–0.0047 PAL) O_2_ estimated by calculating oxygen demand of the 3.0 Ga Jerico Dam paleosol of South Africa, and stability of associated uraninite [42]. An unlikely oxidizing atmosphere of at least 3,000 ppm (0.014 PAL) O_2_ has been proposed for the Jerico Dam paleosol, assuming later chemically reduction by hydrothermal fluids or by burial gleization of organic acids [136]. However, there is no clear hydrothermal enrichment of heavy rare earth elements in the Jericho Dam profile [187]. Furthermore, burial gleization in paleosols of Triassic forest ecosystems much more productive than envisaged for the Archean extends only 30 cm [188], an order of magnitude less than the 3.3 m within the Jerico Dam Paleosol [42, 187]. These later alterations do not apply to the Nsuze paleosols of South Africa, slightly younger at 2.9 Ga, and with a lack of oxidative Cr and U recycling suggestive of 12–63 ppm (0.0006–0.003 PAL) O_2_ in the atmosphere [189]. Estimates of 20–200 ppm (0.00095–0.0095 PAL) O_2_ come from cerium anomalies in the 3.0–3.3 Ga Keonjhar deep weathering remnant of India, although that paleosol shows clear potash metasomatism and hydrothermal heavy rare earth enrichment that might compromise the estimate [137]. Pyrite (FeS), uraninite (UO_2_), siderite (FeCO_3_) and gersdorfffite (NiAsS) are common (57–85%) redox-sensitive minerals in heavy mineral separations from fluvial siliciclastic sediments dated from 3.2–2.7 Ga in the Pilbara region [190]. This result may not apply to banded iron formations in which iron minerals are largely hydrothermal or biogenic [191]. Detrital pyrite could survive atmospheric oxidation by rapid burial in tectonically active regions [192], but this objection is unlikely for the Strelley Pool Formation deposited on a non-volcanic, stable, continental landmass [46]. Low atmospheric O_2_ and consequent lack of an ozone layer in the stratosphere has also been suggested to explain mass-independent fractionation of sulfur isotopes in Archean pyrites and barites of the Pilbara region [153, 154]. Mass-independent isotopic fractionation of sulfur can also be due to chemisorption with organic matter [4] at modest burial temperatures (150–200°C), so a reassessment of organic effects on the mass-independent sulfur-isotopic record is needed.

### Microbiota

The Strelley Pool Formation includes a variety of permineralized microfossils [8, 21–25 150]. Comparable microfossils are even better known in diverse assemblages from the 3.0 Ga Farrel Quarzite near Mt Grant in northeastern Pilbara Shire [26, 193–197], the 3.44 Ga Mt Ada Basalt 4 km west of old Panorama Homestead [48, 125 198, 199], and the 3.46 Ga Apex chert near Marble Bar [10, 11, 89]. Apex chert microfossils have been deformed by neomorphic recrystallization, and so doubted as genuine [8], but their organic matter is biogenic [11, 66, 93, 94], and some microfossils discovered during this work resisted matrix recrystallization (arrows Fig 14B). The Mt Ada microfossils are very well preserved, although there have been problems in relocating the exact site for re-evaluation of their geological context [198]. Cherty alluvial paleosols include a variety of microfossils (Fig 15), not counting those in parent sandstones [21–23] or in stromatolites of the Strelley Pool Formation [23].

Prominent among microfossils in paleosols of the Strelley Pool Formation are large spindles (Fig 15A–15F). These microfossils are considered indigenous to the paleosols because nodules within Jurl paleosols are like those in modern soils taking millennia to coalesce [128], and decay times of microbes under anoxic conditions are less than 160 days [200]. Comparable spindle microfossils also have been found in the 3.4 Ga Kromberg Formation of South Africa [201], 3.2 Ga Clutha Formation of South Africa [202], 3.0 Ga Farrel Quartzite of Western Australia [21, 26, 194], and 1.3 Ga Kendall River Formation of Canada [203, 204]. These microfossil spindles have been identified with a variety of acritarch genera: *Pterospermopsis*, *Pterospermella*, and *Pterospermopsimorpha* [193]. These acritarch genera differ in being double-walled and spherical with narrow flanges, and they either lack projections [205], or have more than two blunt-ending projections [206–208]. The Strelley Pool Formation spindles are often clustered with their ends continuous with filaments that appear to join them in bunches (Fig 15C and 15D), and in addition have small internal bodies (Fig 15E and 15F). *Primoflagella* of the Ediacaran Kotlin Formation of Russia is a somewhat similar microfossil, and has one end tapering to filaments, but the other end differs in being blunt [209]. The Strelley Pool Formation spindles also similar to *Eupoikilofusa cloudii* from the Cambrian Gouhou and Xiamaling Formations of China [210, 211], and *Eupoikilofusa* sp. from the 1.2 Ga Society Cliffs Formation of Baffin Island [212]. Strelley Pool microfossil spindles deserve their own new name, but are provisionally referred here to *Eupoikilofusa* sp. Also found in Strelley Pool paleosols were spheroidal microfossils (Fig 15G), broadly comparable with *Archaeosphaeroides pilbarensis* [198]. Four distinct kinds of Archean spheroids are recognized in the Farrel Quartzite [26, 194], and five in the the Strelley Pool Formation [25], including forms with complex walls (Fig 15G). Comparable spindle microfossils in the 3.0 Ga Farrel Quartzite of Western Australia have been interpreted as sporangia of actinobacteria, and the spheroidal microfossils as sulfur-oxidizing photosynthetic bacteria, and methanogens [26].

Microfossil assemblages of paleosols of the Strelley Pool Formation are quite different from those of marine cherts interbedded with pillow basalts of the Apex Basalt [10–12, 91, 94] and Mt Ada Basalt [125, 198, 199, 213]. Isotopic composition of Apex chert microfossils have been interpreted as evidence for photosynthetic sulfur bacteria, methanogenic Archaeans, and γ-proteobacterial methanotrophs [91]. Apex and Mt Ada Basalt Archean marine assemblages are dominated by septate filaments (*Primaevifilum* [10]), whereas non-marine assemblages described here have distinctive spindles (cf. *Eopoikilofusa* [210]). Both assemblages include spheroids (*Archaeosphaeroides* [198]).

## Conclusions

Paleosols within and below the Strelley Pool Formation reveal many aspects of life and landscapes 3.3 billion years ago (Fig 18). The degree of weathering of the paleosols compared with modern soils [141], reveals temperate mean annual paleotemperatures (between 6.9 and 11.8 ^o^C) as expected for a likely paleolatitude between 20.5 ± 5^o^ for the ca. 3460 Ma upper Apex Chert [147], and 59.0 ± 8.8^o^ for the 2.86 ± 0.2 Ga Millindinna Complex of the north Western Australia [148]. Humid paleoclimate is apparent from chemical composition of the paleosols compared with modern soils [139]: mean annual precipitation of between 1037 and 1552 mm, ± 182 mm for different paleosols. Paleosols beneath the Strelley Pool Formation are clayey and lack salts as expected in humid climate, but evaporite pseudomorphs in paleosols within Strelley Pool Formation give the superficial appearance of arid paleoclimates. The salts were pedogenic barite (BaSO_4_), known in humid soils receiving 1000–1200 mm mean annual precipitation, and formed by local microbially induced precipitation, or redox changes at the water table, independent of paleoclimate [114].

A high level of soil carbonic acid and atmospheric carbon dioxide is apparent from integrated profile depletions of alkali and alkali earth cations normalized for likely time of soil formation of Jurta paleosols [163]: 2011 to 3473 ppm by volume CO_2_ (7.18 to 11.3 preindustrial atmospheric level or PAL of 280 ppmv). Another constraint comes from pseudomorphs of nahcolite (NaHCO_3_) observed in the Wanta paleosols, and prone to recrystallization to trona (Na_3_(CO_3_)(HCO_3_)∙2H_2_O) or natron (Na_2_CO_3_∙10H_2_O) at CO_2_ levels of less than 840 ppm by volume [130]. Archean soil-respired CO_2_ levels are unknown, but may have reached 500–1000 ppmv (1.8–3.6 PAL) like modern salty soils of deserts [162]. Atmospheric CO_2_ then would be the difference including the nahcolite constraint and paleosol estimates, some 945–3616 ppmv (3.4–12.9 PAL). Even if Archean soils were lifeless, such atmospheric levels of CO_2_ are inadequate for a greenhouse capable of maintaining likely temperate Archean paleotemperatures given the faint young sun, and other greenhouse gases are needed, including CH_4_, C_2_H_6_, SO_2_, OCS, and water vapor [4]. Volcanogenic SO_2_ is especially suggested by the abundance of sulphate pseudomorphs in alluvial paleosols of the Strelley Pool Formation (Jurl, Jurnpa and Wanta pedotypes).

Low levels of soil O_2_ can be estimated from pre-Strelley Paleosols by modifying CO_2_ algorithms [163] for profile integrated oxidation of iron in Jurta paleosols: 1124 to 2181 ppm. These can be regarded as a maximum values for the atmosphere, because of likely biological contribution to iron oxidation, not by oxygenic photosynthesis, but by photoferrotrophs also considered responsible for banded iron formations [184]. Although all the paleosols within and below the Strelley Pool Formation are drab colored and have high ferrous to ferric iron ratios (Figs 12 and 13), Wanta profiles at 155 m depth in the Coonterunah Deep drill core [85] and in outcrop near Marble Bar present hybrid paleosols with subsurface sand crystals and nodules like other paleosols (Fig 5D) but also surficial hematite (Fig 7J) laminated like marine and lacustrine banded iron formation [184]. Wanta paleosols may have been episodically waterlogged to allow growth of banded iron formation photoferrotrophs. No hematite was seen in Jurta paleosols at 163.7–170.9 m in the Coonterunah Deep Drill core [85], or in any of the outcrop sections (Figs 2 and 5), but limited photoferrotrophy may have contributed to iron-manganese stain of ped surfaces in these paleosols. These observations of limited atmospheric oxidation do not support the idea of laterite 12–21 m below the Jurta paleosol on Trendall Ridge [4]. These are thin (1–2 m) pods of banded iron formation in the parent Panorama Formation, which is at a low angle (restored to 4^o^ west) with the unconformable Strelley Pool Formation. No laterite was found below the Jurta paleosol or in deeper rocks to 329.8 m in Coonterunah core [85]. Archean hematite may have been formed by iron-oxidizing bacteria in aquatic ecosystems of banded iron formations [184]. Well drained Archean paleosols were drab as expected for an atmosphere very low in free oxygen, but waterlogged Archean soils could be reddened by biogenic hematite, in a redox relationship upside-down compared with soils today.

Microfossils have been widely reported from what are here recognized as paleosols in the Strelley Pool Formation [23, 24, 150], and microfossil traces in pyrite grains from basal sands of the formation may have been redeposited from Jurta paleosols [18, 19]. Microbial sulphate reduction and sulfur disproportionation have both been inferred from sulfur isotopic data from the Strelley Pool Formation [23]. Permineralized microfossils were found within surface horizons of alluvial paleosols, and include spindle like forms [193] comparable with *Eopoikilofusa* [212], and spheroidal forms comparable with *Archaeosphaeroide*s [198], like those other Archean terrestrial communities of sulfur oxidizing bacteria, actinobacteria and methanogens [26]. Communities of microfossils from cherts of presumed marine origin interbedded with pillow basalts of the Mt Ada [198] and Apex Basalts [10, 11] are distinct from those of the Strelley Pool Formation paleosols [23, 24, 150], in dominance by septate filaments (*Primaevifilum*), and included sulfur bacteria, methanotrophs and methanogens [91]. Microfossiliferous Apex chert is a 15 m thick bed conformable with overlying and underlying pillow basalts, and has no hydrothermal features such as flanking phyllitic alteration, stockwork dikes, nor copper-lead-zinc sulfide mineralization. Clay and hydroxide minerals preserved within early diagenetic chert are evidence of moderate burial temperatures (80–150°C [38]), lower than likely regional metamorphic temperatures (350°C [64]). This revised context for Apex chert microfossils supports the sedimentary interpretation [10] rather than the abiotic hydrothermal hypothesis [6]. Poor preservation of many of the microfossils is not necessarily evidence of abiotic origin, but instead evidence for decay, a key biogenic criterion [94].

## Supporting information

S1 File Inclusivity in global research. (DOCX)

S1 Table Grain size of Archean paleosols from point counting (500 points). (DOCX)

S2 Table Mineral content of Archean paleosols from point counting (500 points). (DOCX)

S3 Table Chemical composition (wt %) of Archean paleosols by XRF. (DOCX)

S4 Table Metadata for LA-ICP-MS U-(Th-)Pb analyses. (XLSX)

S5 Table U-Pb isotope ratios and trace element concentrations by LA-ICPMS: Sample data. (XLSX)

S6 Table U-Pb isotope ratios and trace element concentrations by LA-ICPMS: Standard data. (XLSX)

S7 Table CA-TIMS U-Pb isotopic data. (XLSX)

S8 Table Durations of Jurta paleosols using various modern chronofunctions. (DOCX)

S9 Table Durations of paleosols within the Strelley Pool Formation. (DOCX)

S10 Table Gaussian error propagation for atmospheric CO_2_ and O_2_ estimates. (DOCX)

S11 Table Components of Gaussian error quadrature for atmospheric CO_2_ and O_2._ (DOCX)

S12 Table Errors (2σ) on determination of past soil CO_2_ from Jurta paleosols. (DOCX)

S13 Table Errors (2σ) on determination of past soil O_2_ from Jurta paleosols. (DOCX)
