# Supplementary material for: Archean (3.3 Ga) paleosols and paleoenvironments of Western Australia
Source: PLoS One. 2023 Sep 27;18(9):e0291074. doi: 10.1371/journal.pone.0291074 (PMC10530016; doi:10.1371/journal.pone.0291074)
Supplement: S1 Table — (DOCX) [file pone.0291074.s002.docx]

**Table S1. Grain size of Archean paleosols from point counting (500 points)**

| Pedotype | Hor. | No. R- | %gravel | % sand | % silt | % clay | Textural class | Petrography |
| --- | --- | --- | --- | --- | --- | --- | --- | --- |
| Jurl | < | 3781 | 7.4 | 45.6 | 33.8 | 13.2 | Silt loam | Porphyroskelic silasepic |
| Jurl | A | 3782 | 0 | 15.8 | 60.8 | 23.4 | Silt loam | Porphyroskelic silasepic |
| Jurl | C | 3783L | 0 | 7.4 | 71.6 | 21.0 | Silt loam | Porphyroskelic silasepic |
| Jurl | By | 3783U | 0 | 17.4 | 63.8 | 18.8 | Silt loam | Porphyroskelic silasepic |
| Jurta | A | 3784 | 0 | 16.2 | 54.2 | 29.6 | Silty clay loam | Porphyroskelic omnisepic |
| Jurta | A | 3785 | 0 | 15.2 | 54.0 | 30.8 | Silty clay loam | Porphyroskelic omnisepic |
| Jurta | Bw | 3786 | 0 | 22.8 | 46.2 | 31.0 | Clay loam | Porphyroskelic clinobimasepic |
| Jurta | Bw | 3787 | 0 | 22.8 | 51.2 | 26.0 | Silt loam | Porphyroskelic clinobimasepic |
| Jurta | C | 3788 | 0 | 30.8 | 49.6 | 19.4 | Loam | Agglomeroplasmic clinobimasepic |
| Jurta | C | 3789 | 0 | 52.4 | 37.2 | 10.4 | Sandy loam | Intertextic silasepic |
| Jurta | R | 3790 | 0 | 46.0 | 42.0 | 12.0 | Loam | Intertextic insepic |
| Jurta | R | 3791 | 0 | 37.8 | 40.8 | 21.4 | Loam | Intertextic silasepic |
| Jurta | R | 3792U | 0 | 22.2 | 63.0 | 14.8 | Silt loam | Porphyroskelic insepic |
| Jurta | R | 3792L | 0 | 21.2 | 58.8 | 20.0 | Silt loam | Porphyroskelic insepic |
| Wanta | < | 3793 | 5.8 | 63.8 | 26.2 | 4.2 | Sandy loam | Porphyroskelic insepic |
| Wanta | A | 3794 | 0 | 25.8 | 49.2 | 25.0 | Loam | Porphyroskelic insepic |
| Wanta | A | 3795 | 0.2 | 24.4 | 60.2 | 15.2 | Silt loam | Porphyroskelic insepic |
| Wanta | By | 3796 | 0.4 | 27.4 | 57.4 | 14.8 | Silt loam | Agglomeroplasmic insepic |
| Wanta | C | 3797 | 0 | 33.4 | 53.4 | 13.2 | Silt loam | Agglomeroplasmic insepic |
| Wanta | A | 3798 | 0 | 16.6 | 56.0 | 27.4 | Silty clay loam | Porphyroskelic insepic |
| Wanta | By | 3799 | 0 | 16.2 | 60.4 | 23.4 | Silt loam | Porphyroskelic insepic |
| Wanta | By | 3800 | 0.8 | 20.2 | 58.8 | 20.2 | Silt loam | Agglomeroplasmic insepic |
| Wanta | C | 3801 | 0 | 20.0 | 58.4 | 21.6 | Silt loam | Porphyroskelic insepic |
| Jurl | < | 3802 | 4.4 | 42.2 | 33.6 | 19.8 | Loam | Agglomeroplasmic silasepic |
| Jurl | A | 3803 | 0.2 | 24.6 | 45.8 | 29.4 | Clay loam | Porphyroskelic mosepic |
| Jurl | By | 3804 | 0 | 33.2 | 42.4 | 24.4 | Loam | Porphyroskelic insepic |
| Jurl | By | 3805 | 0 | 37.2 | 42.2 | 20.6 | Loam | Porphyroskelic insepic |
| Jurl | C | 3806 | 0 | 39.0 | 41.0 | 20.0 | Loam | Agglomeroplasmic insepic |
| Ngumpu | A | 3807 | 0 | 36.0 | 39.0 | 25.0 | Loam | Porphyroskelic insepic |
| Ngumpu | C | 3808 | 0 | 48.0 | 35.8 | 16.2 | Loam | Agglomeroplasmic skelmosepic |
| Jurta | < | 4202 | 25.0 | 56.2 | 13.8 | 5.0 | Sandy loam | Granular silasepic |
| Jurta | A | 4203 | 0 | 15.2 | 55.8 | 39.0 | Clay | Porphyroskelic omnisepic |
| Jurta | A | 4204 | 0 | 21.2 | 48.4 | 30.4 | Clay loam | Porphyroskelic omnisepic |
| Jurta | Bw | 4205 | 0 | 35.0 | 40.8 | 24.2 | Loam | Porphyroskelic omnisepic |
| Jurta | C | 4206 | 0 | 35.6 | 42.2 | 22.2 | Loam | Agglomeroplasmic clinobimasepic |
| Jurta | C | 4207 | 0 | 42.8 | 57.2 | 0 | Silt loam | Agglomeroplasmic mosepic |
| Jurta | R | 4208 | 0 | 37.2 | 62.8 | 0 | Silt loam | Agglomeroplasmic insepic |
| Jurnpa | < | 4318 | 0 | 60.2 | 34.2 | 5.6 | Sandy loam | Granular silasepic |
| Jurnpa | A | 4319 | 9.0 | 57.6 | 25.2 | 8.2 | Sandy loam | Granular silasepic |
| Jurnpa | A | 4320 | 0.8 | 31.8 | 45.8 | 21.6 | Loam | Agglomeroplasmic silasepic |
| Jurnpa | A | 4321 | 3.8 | 37.0 | 39.0 | 20.2 | Loam | Agglomeroplasmic silasepic |
| Jurnpa | By | 4322 | 22.0 | 30.8 | 32.2 | 15.0 | Loam | Agglomeroplasmic insepic |
| Jurnpa | C | 4323 | 0 | 47.8 | 40.8 | 11.4 | Loam | Granular silasepic |
| Jurta | < | 4502 | 7 | 52.2 | 34.2 | 6.6 | Sandy loam | Granular silasepic |
| Jurta | A | 4503 | 2.6 | 26.0 | 26.0 | 45.4 | Clay | Porphyroskelic clinobimasepic |
| Jurta | A | 4504 | 1.4 | 22.2 | 33.2 | 43.2 | Clay | Porphyroskelic omnisepic |
| Jurta | Bw | 4505 | 2.4 | 18.0 | 35.0 | 44.6 | Clay | Porphyroskelic clinobimasepic |
| Jurta | C | 4506 | 1.8 | 25.0 | 38.2 | 34.0 | Clay loam | Porphyroskelic clinobimasepic |
| Jurta | R | 4507 | 1.4 | 59.0 | 13.4 | 26.2 | Sandy clay loam | Porphyroskelic clinobimasepic |
| Jurta | R | 4508 | 1.2 | 52.2 | 22.8 | 23.8 | Sandy clay loam | Porphyroskelic mosepic |
