# Supplementary material for: Archean (3.3 Ga) paleosols and paleoenvironments of Western Australia
Source: PLoS One. 2023 Sep 27;18(9):e0291074. doi: 10.1371/journal.pone.0291074 (PMC10530016; doi:10.1371/journal.pone.0291074)
Supplement: S2 Table — (DOCX) [file pone.0291074.s003.docx]

**Table S2. Mineral content of Archean paleosols from point counting (500 points)**

| Pedo-type | Hor-izon | No. R- | % clay | % alunite | % evaporite | % pyroxene | % rock fragment | % feldspar | % mica | % pyrophyllite | % quartz | % opaque |
| --- | --- | --- | --- | --- | --- | --- | --- | --- | --- | --- | --- | --- |
| Jurl | < | 3781 | 13.0 | 0 | 3.2 | 0 | 25.4 | 29.6 | 0.6 | 0 | 23.0 | 5.2 |
| Jurl | A | 3782 | 23.6 | 0 | 0 | 0 | 3.4 | 39.8 | 2.2 | 0 | 28.0 | 3.0 |
| Jurl | C | 3783U | 20.0 | 0 | 6.6 | 0 | 4.2 | 39.2 | 3.8 | 0 | 21.8 | 4.4 |
| Jurl | By | 3783L | 21.8 | 0 | 2.4 | 0 | 6.4 | 44.0 | 3.2 | 0 | 20.0 | 2.2 |
| Jurta | A | 3784 | 32.3 | 0 | 0 | 0 | 6.4 | 25.0 | 4.8 | 9.6 | 9.4 | 12.6 |
| Jurta | A | 3785 | 31.0 | 0 | 0 | 0 | 5.4 | 21.4 | 6.0 | 10.0 | 11.0 | 15.2 |
| Jurta | Bw | 3786 | 32.4 | 0 | 0 | 0 | 8.8 | 19.2 | 5.0 | 17.2 | 9.4 | 8.0 |
| Jurta | Bw | 3787 | 28.4 | 0 | 0 | 0 | 7.0 | 19.8 | 5.0 | 17.0 | 9.4 | 7.0 |
| Jurta | C | 3788 | 21.2 | 0 | 0 | 0 | 6.0 | 29.6 | 1.4 | 23.4 | 12.0 | 6.4 |
| Jurta | C | 3789 | 10.6 | 0 | 0 | 0 | 7.4 | 37.0 | 8.6 | 10.8 | 12.6 | 13.0 |
| Jurta | R | 3790 | 13.2 | 0 | 0 | 9.6 | 24.0 | 23.8 | 4.0 | 10.2 | 6.4 | 8.8 |
| Jurta | R | 3791 | 0 | 0 | 0 | 7.4 | 23.0 | 48.2 | 2.2 | 7.6 | 0 | 11.4 |
| Jurta | R | 3792U | 15.8 | 0 | 0.2 | 0 | 3.4 | 39.8 | 0 | 9.6 | 28.0 | 3.2 |
| Jurta | R | 3792L | 18.8 | 0 | 2.6 | 0 | 8.4 | 38.4 | 0 | 5.4 | 22.6 | 3.8 |
| Wanta | < | 3793 | 10.4 | 0 | 0 | 0 | 36.8 | 28.2 | 0.4 | 0 | 19.6 | 4.6 |
| Wanta | A | 3794 | 24.2 | 0 | 0 | 0 | 2.6 | 39.4 | 0.4 | 0 | 29.0 | 4.4 |
| Wanta | A | 3795 | 17.4 | 0 | 3.2 | 0 | 2.4 | 40.2 | 1.6 | 0 | 32.4 | 2.8 |
| Wanta | By | 3796 | 14.6 | 0 | 16.4 | 0 | 3.8 | 34.2 | 2.6 | 0 | 21.2 | 7.0 |
| Wanta | C | 3797 | 13.2 | 0 | 5.6 | 0 | 21.0 | 29.2 | 2.0 | 0 | 20.6 | 8.4 |
| Wanta | A | 3798 | 28.6 | 0 | 2.8 | 0 | 2.8 | 33.2 | 1.4 | 0 | 23.0 | 8.6 |
| Wanta | By | 3799 | 21.0 | 0 | 5.4 | 0 | 3.8 | 37.2 | 2.6 | 0 | 28.2 | 1.8 |
| Wanta | By | 3800 | 21.6 | 0 | 10.6 | 0 | 5.2 | 32.0 | 5.2 | 0 | 22.4 | 3.0 |
| Wanta | C | 3801 | 21.8 | 0 | 0 | 0 | 13.4 | 28.2 | 7.8 | 0 | 24.6 | 4.2 |
| Jurl | < | 3802 | 20.8 | 0 | 0 | 0 | 23.8 | 24.0 | 2.6 | 0 | 20.2 | 8.4 |
| Jurl | A | 3803 | 32.0 | 0 | 1.8 | 0 | 4.4 | 32.3 | 2.2 | 0 | 21.0 | 6.4 |
| Jurl | By | 3804 | 23.6 | 0 | 4.8 | 0 | 8.0 | 33.2 | 2.4 | 0 | 22.4 | 5.6 |
| Jurl | By | 3805 | 21.4 | 0 | 7.2 | 0 | 23.4 | 21.8 | 2.2 | 0 | 16.4 | 7.6 |
| Jurl | C | 3806 | 20.4 | 0 | 1.6 | 0 | 29.2 | 22.4 | 2.8 | 0 | 17.6 | 6.0 |
| Ngumpu | A | 3807 | 23.4 | 0 | 0 | 0 | 19.8 | 25.8 | 3.6 | 0 | 22.4 | 5.0 |
| Ngumpu | C | 3808 | 17.2 | 0 | 0 | 0 | 34.4 | 20.2 | 4.8 | 0 | 15.8 | 7.6 |
| Jurta | < | 4202 | 5.8 | 0 | 0 | 0 | 11.6 | 41.2 | 1.2 | 0 | 38.6 | 1.6 |
| Jurta | A | 4203 | 27.8 | 0 | 0 | 0 | 14.2 | 15.2 | 1.2 | 16.6 | 11.2 | 13.8 |
| Jurta | A | 4204 | 30.6 | 0 | 0 | 0 | 5.4 | 27.6 | 2.2 | 7.2 | 7.6 | 10.8 |
| Jurta | Bw | 4205 | 24.0 | 0 | 0 | 0 | 6.6 | 36.0 | 5.4 | 4.2 | 12.0 | 11.8 |
| Jurta | C | 4206 | 14.4 | 0 | 0 | 0 | 1.4 | 36.2 | 11.0 | 20.2 | 5.0 | 11.8 |
| Jurta | C | 4207 | 0 | 0 | 0 | 0 | 37.2 | 38.8 | 3.4 | 10.0 | 0 | 10.6 |
| Jurta | R | 4208 | 0 | 0 | 0 | 0 | 38.4 | 25.6 | 8.2 | 11.0 | 0 | 16.8 |
| Jurnpa | < | 4318 | 5.6 | 0 | 0 | 0 | 6.0 | 42.4 | 2.2 | 0 | 37.8 | 6.0 |
| Jurnpa | < | 4319 | 5.6 | 0 | 0.6 | 0 | 11.6 | 40.6 | 1.6 | 0 | 37.6 | 3.0 |
| Jurnpa | A | 4320 | 19.0 | 0 | 0.6 | 0 | 7.8 | 34.2 | 1.6 | 0 | 31.8 | 4.8 |
| Jurnpa | A | 4321 | 19.8 | 0 | 2.8 | 0 | 15.4 | 29.0 | 1.6 | 0 | 23.8 | 7.4 |
| Jurnpa | By | 4322 | 13.0 | 7.4 | 25.8 | 0 | 3.0 | 20.4 | 1.0 | 0 | 19.8 | 9.6 |
| Jurnpa | C | 4323 | 12.8 | 0 | 0 | 0 | 9.4 | 34.6 | 2.4 | 0 | 35.6 | 5.0 |
| Jurta | < | 4502 | 6.8 | 9.0 | 0 | 0 | 0.8 | 41.8 | 0.4 | 0 | 37.4 | 3.8 |
| Jurta | A | 4503 | 30.3 | 0 | 0 | 0 | 19.1 | 11.7 | 0 | 21.3 | 9.1 | 8.1 |
| Jurta | A | 4504 | 30.8 | 0 | 0.6 | 0 | 10.6 | 20.8 | 1.6 | 12.4 | 16.6 | 6.6 |
| Jurta | Bw | 4505 | 31.6 | 0 | 0 | 0 | 11.0 | 16.0 | 0.8 | 12.4 | 14.0 | 14.2 |
| Jurta | C | 4506 | 14.0 | 0 | 1.0 | 0 | 31.2 | 9.6 | 0.2 | 21.8 | 9.8 | 12.4 |
| Jurta | R | 4507 | 3.2 | 0 | 0 | 0 | 51.4 | 3.0 | 0.8 | 22.2 | 3.4 | 16.0 |
| Jurta | R | 4508 | 2.6 | 0 | 0 | 0 | 50.6 | 9.6 | 0 | 24.2 | 6.6 | 6.4 |
