# Supplementary material for: Archean (3.3 Ga) paleosols and paleoenvironments of Western Australia
Source: PLoS One. 2023 Sep 27;18(9):e0291074. doi: 10.1371/journal.pone.0291074 (PMC10530016; doi:10.1371/journal.pone.0291074)
Supplement: S3 Table — (DOCX) [file pone.0291074.s004.docx]

**Table S3. Chemical composition (wt %) of Archean paleosols by XRF**.

| Pedotype | Hor. | No. | SiO_2_ | TiO_2_ | Al_2_O_3_ | Fe_2_O_3_ | FeO | MnO | MgO | CaO | Na_2_O | K_2_O | Cr_2_O_3_ | P_2_O_5_ | SrO | BaO | LOI | Total | g.cm^-3^ |
| --- | --- | --- | --- | --- | --- | --- | --- | --- | --- | --- | --- | --- | --- | --- | --- | --- | --- | --- | --- |
| Jurl | A | 3782 | 97.51 | 0.06 | 0.31 | 1.58 | 1.41 | 0.01 | 0.02 | 0.02 | 0.02 | 0.07 | 0.01 | 0.003 | <0.01 | 0.01 | -0.2 | 99.41 | 2.65 |
| Jurl | By | 3783 | 97.07 | 0.04 | 0.66 | 2.56 | 1.91 | 0.02 | 0.03 | 0.01 | 0.04 | 0.14 | 0.01 | 0.005 | <0.01 | 0.01 | -0.39 | 100.2 | 2.57 |
| Jurta | A | 3784 | 76.87 | 0.98 | 13.89 | 2.15 | 1.67 | 0.02 | 0.08 | 0.05 | 0.77 | 2.70 | 0.05 | 0.008 | 0.01 | 0.29 | 1.71 | 99.58 | 2.69 |
| Jurta | Bw | 3785 | 75.04 | 1.08 | 15.76 | 0.84 | 0.64 | 0.01 | 0.07 | 0.04 | 0.70 | 3.36 | 0.05 | 0.012 | 0.01 | 0.39 | 2.17 | 99.52 | 2.66 |
| Jurta | Bw | 3786 | 76.25 | 1.02 | 14.99 | 0.50 | 0.51 | <0.01 | 0.07 | 0.02 | 0.69 | 3.16 | 0.05 | 0.008 | <0.01 | 0.33 | 2.13 | 99.22 | 2.71 |
| Jurta | Bw | 3787 | 73.76 | 1.16 | 16.88 | 0.91 | 0.7 | <0.01 | 0.07 | 0.03 | 0.82 | 3.52 | 0.05 | 0.009 | 0.01 | 0.37 | 2.30 | 99.89 | 2.65 |
| Jurta | C | 3788 | 74.7 | 1.18 | 16.43 | 0.70 | 0.64 | <0.01 | 0.08 | 0.03 | 0.80 | 3.39 | 0.05 | 0.016 | 0.01 | 0.38 | 2.28 | 100.05 | 2.73 |
| Jurta | C | 3789 | 78.16 | 1.01 | 14.37 | 0.75 | 0.45 | <0.01 | 0.11 | 0.04 | 1.16 | 2.12 | 0.05 | 0.01 | 0.01 | 0.15 | 2.21 | 100.15 | 2.66 |
| Jurta | C | 3790 | 63.51 | 0.93 | 13.87 | 12.17 | 9.86 | 0.02 | 3.84 | 0.02 | 0.70 | 0.44 | 0.05 | 0.012 | <0.01 | 0.03 | 3.93 | 99.53 | 2.80 |
| Jurta | R | 3791 | 56.98 | 0.89 | 13.83 | 13.86 | 11.3 | 0.08 | 4.94 | 0.05 | 0.44 | 0.40 | 0.05 | 0.015 | <0.01 | 0.02 | 8.10 | 99.66 | 2.78 |
| Jurta | R | 3792 | 94.43 | 0.08 | 1.56 | 2.62 | 2.42 | 0.02 | 0.17 | 0.01 | 0.08 | 0.25 | 0.01 | 0.005 | <0.01 | 0.05 | -0.01 | 99.26 | 2.64 |
| Wanta | A | 3794 | 96.61 | 0.03 | 0.50 | 1.58 | 1.28 | 0.01 | 0.17 | 0.02 | 0.03 | 0.04 | 0.03 | 0.002 | <0.01 | <0.01 | 0.08 | 99.06 | 2.63 |
| Wanta | A | 3795 | 97.39 | 0.02 | 0.22 | 2.22 | 1.66 | 0.02 | 0.03 | 0.01 | 0.02 | 0.04 | 0.02 | 0.002 | <0.01 | <0.01 | -0.33 | 99.64 | 2.63 |
| Wanta | By | 3796 | 98.94 | 0.01 | 0.13 | 0.87 | 0.7 | <0.01 | 0.03 | 0.03 | 0.01 | 0.02 | 0.01 | 0.002 | <0.01 | <0.01 | 0.08 | 100.15 | 2.64 |
| Wanta | C | 3797 | 97.99 | 0.01 | 0.16 | 1.33 | 0.89 | 0.01 | 0.02 | 0.01 | 0.02 | 0.04 | 0.01 | 0.003 | <0.01 | <0.01 | -0.07 | 99.52 | 2.63 |
| Wanta | A | 3798 | 97.12 | 0.01 | 0.08 | 0.85 | 0.64 | <0.01 | 0.02 | 0.01 | 0.01 | 0.03 | 0.01 | 0.002 | <0.01 | <0.01 | 0.06 | 98.17 | 2.63 |
| Wanta | By | 3799 | 97.68 | 0.03 | 0.33 | 1.89 | 1.6 | 0.02 | 0.02 | 0.06 | 0.02 | 0.06 | 0.03 | 0.007 | <0.01 | 0.01 | -0.12 | 100 | 2.61 |
| Wanta | By | 3800 | 97.56 | 0.03 | 0.18 | 1.01 | 0.83 | 0.01 | 0.02 | 0.04 | 0.01 | 0.03 | 0.03 | 0.003 | <0.01 | <0.01 | -0.01 | 98.87 | 2.53 |
| Wanta | C | 3801 | 97.19 | 0.02 | 0.46 | 2.12 | 1.66 | 0.02 | 0.02 | 0.01 | 0.01 | 0.10 | 0.02 | 0.004 | <0.01 | 0.04 | -0.12 | 99.88 | 2.62 |
| Jurl | A | 3803 | 97.49 | 0.04 | 0.50 | 0.93 | 0.7 | 0.01 | 0.01 | 0.01 | 0.01 | 0.14 | 0.04 | 0.004 | <0.01 | 0.06 | 0.26 | 99.46 | 2.61 |
| Jurl | By | 3804 | 96.63 | 0.04 | 0.78 | 1.77 | 1.28 | 0.01 | 0.02 | 0.01 | 0.02 | 0.19 | 0.04 | 0.003 | <0.01 | 0.03 | 0.06 | 99.57 | 2.60 |
| Jurl | By | 3805 | 96.87 | 0.04 | 0.95 | 0.82 | 0.51 | <0.01 | 0.02 | 0.01 | 0.03 | 0.20 | 0.04 | 0.002 | <0.01 | 0.07 | 0.18 | 99.21 | 2.60 |
| Jurl | C | 3806 | 95.8 | 0.04 | 0.78 | 1.99 | 1.72 | 0.01 | 0.02 | 0.01 | 0.02 | 0.17 | 0.04 | 0.004 | <0.01 | 0.06 | -0.12 | 98.78 | 2.62 |
| Ngumpu | A | 3807 | 97.02 | 0.04 | 0.73 | 0.89 | 0.45 | <0.01 | 0.02 | 0.01 | 0.02 | 0.16 | 0.02 | 0.004 | <0.01 | 0.05 | 0.22 | 99.19 | 2.60 |
| Ngumpu | C | 3808 | 96.51 | 0.04 | 0.69 | 1.39 | 1.15 | 0.01 | 0.01 | 0.01 | 0.03 | 0.15 | 0.01 | 0.003 | <0.01 | 0.03 | 0.05 | 98.93 | 2.62 |
| Jurta | A | 4203 | 83.52 | 0.33 | 10.38 | 0.65 | 0.45 | <0.01 | 0.24 | 0.02 | 0.06 | 3.14 | 0.10 | 0.01 | <0.01 | 0.01 | 1.63 | 100.1 | 2.69 |
| Jurta | A | 4204 | 82.88 | 0.36 | 10.58 | 0.95 | 0.64 | <0.01 | 0.22 | 0.01 | 0.06 | 3.22 | 0.11 | 0.005 | <0.01 | 0.01 | 1.60 | 100 | 2.65 |
| Jurta | C | 4205 | 81.40 | 0.39 | 11.63 | 0.62 | 0.38 | <0.01 | 0.27 | 0.02 | 0.05 | 3.30 | 0.10 | 0.021 | <0.01 | 0.02 | 1.99 | 99.81 | 2.67 |
| Jurta | C | 4206 | 73.64 | 0.45 | 16.00 | 1.47 | 0.58 | <0.01 | 0.35 | 0.02 | 0.08 | 4.80 | 0.13 | 0.06 | <0.01 | 0.03 | 2.52 | 99.55 | 2.64 |
| Jurta | R | 4207 | 83.5 | 0.35 | 10.40 | 0.61 | 0.45 | <0.01 | 0.24 | 0.01 | 0.05 | 3.14 | 0.10 | 0.011 | <0.01 | 0.02 | 1.63 | 100.05 | 2.62 |
| Jurta | R | 4208 | 81.95 | 0.32 | 10.26 | 2.6 | 0.77 | <0.01 | 0.23 | 0.01 | 0.05 | 2.79 | 0.08 | 0.039 | <0.01 | 0.01 | 1.98 | 100.3 | 2.62 |
| Jurnpa | < | 4319 | 96.31 | 0.009 | 0.12 | 3.75 | 3.32 | 0.04 | 0.02 | 0.01 | 0.009 | 0.02 | <0.01 | 0.011 | <0.01 | 0.01 | -0.88 | 99.39 | 2.57 |
| Jurnpa | A | 4320 | 96.48 | 0.009 | 0.10 | 2.01 | 1.65 | 0.02 | 0.02 | 0.01 | 0.009 | 0.01 | <0.01 | 0.011 | <0.01 | 0.01 | -0.41 | 98.23 | 2.53 |
| Jurnpa | By | 4321 | 96.06 | 0.009 | 0.13 | 4.17 | 3.39 | 0.04 | 0.02 | 0.01 | 0.009 | 0.01 | <0.01 | 0.011 | <0.01 | 0.01 | -0.99 | 99.48 | 2.47 |
| Jurnpa | By | 4322 | 94.19 | 0.009 | 0.08 | 5.99 | 4.21 | 0.05 | 0.03 | 0.03 | 0.009 | 0.01 | <0.01 | 0.02 | <0.01 | 0.01 | -1.06 | 99.34 | 2.63 |
| Jurnpa | C | 4323 | 97.2 | 0.009 | 0.06 | 1.68 | 1.53 | 0.02 | 0.02 | 0.01 | 0.009 | 0.009 | <0.01 | 0.011 | <0.01 | <0.01 | -0.32 | 98.66 | 2.55 |
| Jurta | A | 4503 | 87.32 | 0.17 | 6.10 | 2.8 | 2.6 | 0.03 | 0.17 | 0.009 | 0.10 | 1.60 | 0.02 | 0.011 | <0.01 | 0.16 | 0.26 | 98.73 | 2.68 |
| Jurta | A | 4504 | 80.82 | 0.33 | 11.44 | 1.14 | 1.02 | 0.01 | 0.31 | 0.009 | 0.19 | 2.98 | 0.03 | 0.012 | <0.01 | 0.29 | 1.47 | 99.03 | 2.68 |
| Jurta | C | 4505 | 81.64 | 0.32 | 11.56 | 0.56 | 0.38 | 0.01 | 0.29 | 0.009 | 0.19 | 3.04 | 0.03 | 0.01 | <0.01 | 0.26 | 1.62 | 99.52 | 2.74 |
| Jurta | C | 4506 | 83.23 | 0.28 | 9.88 | 1.04 | 0.83 | 0.01 | 0.38 | 0.01 | 0.16 | 2.51 | 0.03 | 0.012 | <0.01 | 0.18 | 1.34 | 99.06 | 2.64 |
| Jurta | C | 4507 | 78.15 | 0.38 | 13.12 | 0.78 | 0.57 | 0.01 | 0.44 | 0.009 | 0.2 | 3.46 | 0.04 | 0.012 | <0.01 | 0.16 | 1.84 | 98.58 | 2.54 |
| Jurta | R | 4508 | 85.74 | 0.21 | 7.79 | 0.77 | 0.64 | 0.02 | 0.47 | 0.05 | 0.13 | 1.86 | 0.02 | 0.011 | <0.01 | 0.17 | 1.37 | 98.61 | 2.64 |
| Error |  | All | 2.705 |  | 0.825 | 0.395 |  |  | 0.22 | 0.18 | 0.11 | 0.13 |  | 0.035 |  |  | 0.35 |  | 0.03 |
