# Supplementary material for: Archean (3.3 Ga) paleosols and paleoenvironments of Western Australia
Source: PLoS One. 2023 Sep 27;18(9):e0291074. doi: 10.1371/journal.pone.0291074 (PMC10530016; doi:10.1371/journal.pone.0291074)
Supplement: S8 Table — (DOCX) [file pone.0291074.s009.docx]

**Table S8. Durations of Jurta paleosols using various modern chronofunctions**

| Location | Decom-pacted profile thick-ness (cm) | Decom-pacted solum thick-ness (cm) | Total profile clay (g.cm^-2^) | Age from profile thickness (44.7±11.6 m/Ma rate of Ma et al. 2010) ±24609 years | Age from profile thickness (Lindeburg et al., 2013) ±23192 years | Age from profile thickness (Heimsath et al., 2009) ±5486 years | Age from solum thickness (Markewich et al., 1990) ±190883 years | Age from profile clay (Markewich et al., 1990) ±112817 years |
| --- | --- | --- | --- | --- | --- | --- | --- | --- |
| Strelley Pool | 620 | 465 | 372 | 138,724 | 267,179 | 629,024 | 1,915,770 | 2,457,486 |
| Trendall Ridge | 892 | 639 | 397 | 199,416 | 393,414 | 910,436 | 2,798,437 | 2,631,269 |
| Marble Bar | 717 | 426 | 398 | 95,373 | 177,011 | 428,016 | 1,719,622 | 2,536,530 |

*Note: Thickness of solum is from top to lowest compacted cracks, thickness of profile is from top to fresh jointed rock below zone of corestones (ie base of saprolite). Decompaction is from equation of Sheldon and Retallack (2001) in Table S4 for overburden of 13.826 km.*
