# Supplementary material for: Archean (3.3 Ga) paleosols and paleoenvironments of Western Australia
Source: PLoS One. 2023 Sep 27;18(9):e0291074. doi: 10.1371/journal.pone.0291074 (PMC10530016; doi:10.1371/journal.pone.0291074)
Supplement: S9 Table — (DOCX) [file pone.0291074.s010.docx]

**Table S9. Durations of paleosols within the Strelley Pool Formation**

| Pedotype | Location | Level (m) | Nodule size (cm) | Age (yr) from nodule size | Nodule area (%) | Age (yr) from nodule area |
| --- | --- | --- | --- | --- | --- | --- |
| Jurl | Strelley Pool | 45.3 | 0.7 | 3472 | 9 | 41657 |
| Jurl | Strelley Pool | 45.7 | 0.8 | 3634 | 9 | 41657 |
| Jurl | Strelley Pool | 46.2 | 0.9 | 3782 | 10 | 45644 |
| Jurl | Strelley Pool | 46.7 | 1 | 3920 | 11 | 49631 |
| Jurl | Steer Ridge | 42.6 | 0.8 | 3634 | 8 | 37670 |
| Jurl | Steer Ridge | 42.8 | 0.9 | 3782 | 8 | 37670 |
| Jurl | Steer Ridge | 43 | 1 | 3920 | 9 | 41657 |
| Jurl | Trendall Ridge | 41.5 | 0.9 | 3782 | 10 | 45644 |
| Jurl | Trendall Ridge | 42 | 0.8 | 3634 | 7 | 33683 |
| Jurl | Trendall Ridge | 42.5 | 0.9 | 3782 | 5 | 25709 |
| Jurl | Marble Bar | 20.3 | 0.6 | 3295 | 6 | 29696 |
| Jurl | Marble Bar | 29.7 | 1.1 | 4049 | 10 | 45644 |
| Jurl | Marble Bar | 30 | 1.3 | 4286 | 12 | 53618 |
| Jurl | Marble Bar | 31.8 | 1.3 | 4286 | 10 | 45644 |
| Jurl | Marble Bar | 32.4 | 1.2 | 4171 | 11 | 49631 |
| Jurl | Marble Bar | 39.3 | 1 | 3920 | 10 | 45644 |
| Jurl | Streckfuss | 1.8 | 0.9 | 3782 | 9 | 41657 |
| Jurl | Streckfuss | 2.3 | 1.1 | 4049 | 11 | 49631 |
| Jurnpa | Streckfuss | 1 | 0.8 | 3634 | 6 | 29696 |
| Jurnpa | Streckfuss | 1.7 | 2.1 | 5045 | 5 | 25709 |
| Jurnpa | Trendall Ridge | 43.9 | 1.2 | 4171 | 8 | 37670 |
| Wanta | Marble Bar | 35.7 | 0.8 | 3634 | 9 | 41657 |
| Wanta | Marble Bar | 36.3 | 0.9 | 3782 | 10 | 45644 |
| Wanta | Marble Bar | 36.8 | 0.7 | 3472 | 9 | 41657 |
| Wanta | Marble Bar | 39.9 | 0.6 | 3295 | 8 | 37670 |
| Wanta | Marble Bar | 40.3 | 0.7 | 3472 | 8 | 37670 |
| Wanta | Marble Bar | 40.6 | 0.8 | 3634 | 10 | 45644 |
| Wanta | Marble Bar | 42.2 | 0.7 | 3472 | 8 | 37670 |
| Wanta | Marble Bar | 42.4 | 0.9 | 3782 | 7 | 33683 |
|  |  |  |  |  |  |  |

*Note: Durations were calculated using transfer functions for area of gysum and size of caliche nodules from Table S4.*
