# Supplementary material for: Archean (3.3 Ga) paleosols and paleoenvironments of Western Australia
Source: PLoS One. 2023 Sep 27;18(9):e0291074. doi: 10.1371/journal.pone.0291074 (PMC10530016; doi:10.1371/journal.pone.0291074)
Supplement: S10 Table — (DOCX) [file pone.0291074.s011.docx]

**Table S10. Gaussian error propagation for atmospheric CO_2_ and O_2_ estimates**

| Equations and error (±1σ) |
| --- |
| ${pCO}_{2}=\frac{M}{A\left\lfloor\frac{K_{{CO}_{2}.}P}{1000}+\kappa\frac{D_{{CO}_{2}}.\alpha}{L} \right\rfloor}$ |
| $S_{\bar{{pCO}_{2}}}=\sqrt[2]{\left( \frac{\partial p{CO}_{2}}{\partial M}.S_{\bar{M}} \right)^{2}+\left( \frac{\partial p{CO}_{2}}{\partial A}.S_{\bar{A}} \right)^{2}+\left( \frac{\partial{pCO}_{2}}{\partial K_{{CO}_{2}}}.S_{\bar{K_{{CO}_{2}}}} \right)^{2}+\left( \frac{\partial{pCO}_{2}}{\partial P}.S_{\bar{P}} \right)^{2}+\left( \frac{\partial p{CO}_{2}}{\partial D_{{CO}_{2}}}.S_{\bar{D_{{CO}_{2}}}} \right)^{2}+\left( \frac{\partial p{CO}_{2}}{\partial\alpha}.S_{\bar{\alpha}} \right)^{2}}$ |
| ${pO}_{2}=\frac{M}{A\left\lfloor\frac{K_{O_{2}}P}{1000}+\kappa\frac{D_{O_{2}}\alpha}{L} \right\rfloor}$ |
| $S_{p\bar{O_{2}}=}\sqrt[2]{\left( \frac{\partial{pO}_{2}}{\partial M}.S_{\bar{M}} \right)^{2}+\left( \frac{\partial pO_{2}}{\partial A}.S_{\bar{A}} \right)^{2}+\left( \frac{\partial pO_{2}}{\partial K_{O_{2}}}.S_{\bar{K_{{CO}_{2}}}} \right)^{2}+\left( \frac{\partial pO_{2}}{\partial P}.S_{\bar{P}} \right)^{2}+\left( \frac{\partial pO_{2}}{\partial D_{{CO}_{2}}}.S_{\bar{D_{{CO}_{2}}}} \right)^{2}+\left( \frac{\partial pO_{2}}{\partial\alpha}.S_{\bar{\alpha}} \right)^{2}}$ |
