# Supplementary material for: Archean (3.3 Ga) paleosols and paleoenvironments of Western Australia
Source: PLoS One. 2023 Sep 27;18(9):e0291074. doi: 10.1371/journal.pone.0291074 (PMC10530016; doi:10.1371/journal.pone.0291074)
Supplement: S11 Table — (DOCX) [file pone.0291074.s012.docx]

**Table S11. Components of Gaussian error quadrature for atmospheric CO_2_ and O_2_**

| Explanation | Formula | Partial derivatives |
| --- | --- | --- |
| Combined alkali-earth mass transfer (*M*) | $M=A.{pCO}_{2}\left\lfloor\frac{{P.K}_{{CO}_{2}}}{1000}+\frac{{\kappa.\alpha.D}_{{CO}_{2}}}{L} \right\rfloor$ | $\frac{\partial p{CO}_{2}}{\partial M}=A\left[ \frac{P.K_{{CO}_{2}}}{1000}+\frac{\kappa.\alpha.D_{{CO}_{2}}}{L} \right]$ |
| Duration of soil formation (*A*) | $A=\frac{M}{{pCO}_{2}\left\lfloor\frac{{P.K}_{{CO}_{2}}}{1000}+\frac{{\kappa.\alpha.D}_{{CO}_{2}}}{L} \right\rfloor}$ | $\frac{\partial{pCO}_{2}}{\partial A}=-\frac{M}{\left( {pCO}_{2} \right)^{2}\left[ \frac{{P.K}_{{CO}_{2}}}{1000}+\frac{{\kappa.\alpha.D}_{{CO}_{2}}}{L} \right]}$ |
| Henry’s Law constant ($K_{{CO}_{2}}$) | $K_{{CO}_{2}}=\frac{1000}{P}\left( \frac{M}{A.{pCO}_{2}}-\frac{\kappa.\alpha.D_{{CO}_{2}}}{L} \right)$ | $\frac{\partial p{CO}_{2}}{\partial K_{{CO}_{2}}}=-\frac{1000M}{P.\left( {pCO}_{2} \right)^{2}}$ |
| Mean annual precipitation (*P*) | $P=\frac{1000}{K_{{CO}_{2}}}\left( \frac{M}{A.{pCO}_{2}}-\frac{\kappa.\alpha.D_{{CO}_{2}}}{L} \right)$ | $\frac{\partial{pCO}_{2}}{\partial P}=-\frac{1000M}{K_{{CO}_{2}}.A.\left( {pCO}_{2} \right)^{2}}$ |
| Diffusion constant of CO_2_ in air ($D_{{co}_{2}}$) | $D_{{CO}_{2}}=\frac{L}{\kappa.\alpha}\left( \frac{M}{{A.pCO}_{2}}-\frac{P.K_{{CO}_{2}}}{1000} \right)$ | $\frac{\partial{pCO}_{2}}{\partial D_{{CO}_{2}}}=-\frac{L.M}{\kappa.\alpha.\left( {pCO}_{2} \right)^{2}}$ |
| Ratio of CO_2_ diffusion soil/air ($\alpha)$ | $\alpha=\frac{L}{\kappa.D_{{CO}_{2}}}\left( \frac{M}{{A.pCO}_{2}}-\frac{P.K_{{CO}_{2}}}{1000} \right)$ | $\frac{\partial{pCO}_{2}}{\partial\alpha}=-\frac{L.M}{D_{{CO}_{2}}.\kappa.A.\left( {pCO}_{2} \right)^{2}}$ |
| Iron mass transfer (*F*) | $F={A.pO}_{2}\left\lfloor\frac{{P.K}_{O_{2}}}{1000}+\frac{{\kappa.\alpha.D}_{O_{2}}}{L} \right\rfloor$ | $\frac{\partial pO_{2}}{\partial F}=A\left[ \frac{P.K_{O_{2}}}{1000}+\frac{\kappa.\alpha.D_{O_{2}}}{L} \right]$ |
| Duration of soil formation (*A*) | $A=\frac{F}{{pO}_{2}\left\lfloor\frac{{P.K}_{O_{2}}}{1000}+\frac{{\kappa.\alpha.D}_{O_{2}}}{L} \right\rfloor}$ | $\frac{\partial{pO}_{2}}{\partial A}=-\frac{F}{\left( {pO}_{2} \right)^{2}\left[ \frac{{P.K}_{O_{2}}}{1000}+\frac{{\kappa.\alpha.D}_{O_{2}}}{L} \right]}$ |
| Henry’s Law constant ($K_{O_{2}}$) | $K_{O_{2}}=\frac{1000}{P}\left( \frac{F}{A.{pO}_{2}}-\frac{\kappa.\alpha.D_{O_{2}}}{L} \right)$ | $\frac{\partial pO_{2}}{\partial K_{O_{2}}}=-\frac{1000F}{P.\left( {pO}_{2} \right)^{2}}$ |
| Mean annual precipitation (*P*) | $P=\frac{1000}{K_{O_{2}}}\left( \frac{F}{A.{pO}_{2}}-\frac{\kappa.\alpha.D_{O_{2}}}{L} \right)$ | $\frac{\partial{pO}_{2}}{\partial P}=-\frac{1000F}{K_{O_{2}}.A.\left( {pO}_{2} \right)^{2}}$ |
| Diffusion constant of O_2_ in air ($D_{o_{2}}$) | $D_{O_{2}}=\frac{L}{\kappa.\alpha}\left( \frac{F}{{A.pO}_{2}}-\frac{P.K_{O_{2}}}{1000} \right)$ | $\frac{\partial{pO}_{2}}{\partial D_{O_{2}}}=-\frac{L.F}{\kappa.\alpha.\left( {pO}_{2} \right)^{2}}$ |
| Ratio of O_2_ diffusion soil/air ($\alpha)$ | $\alpha=\frac{L}{\kappa.D_{{CO}_{2}}}\left( \frac{F}{{A.pO}_{2}}-\frac{P.K_{{CO}_{2}}}{1000} \right)$ | $\frac{\partial{pO}_{2}}{\partial\alpha}=-\frac{L.F}{D_{O_{2}}.\kappa.A.\left( {pO}_{2} \right)^{2}}$ |
