# Supplementary material for: Archean (3.3 Ga) paleosols and paleoenvironments of Western Australia
Source: PLoS One. 2023 Sep 27;18(9):e0291074. doi: 10.1371/journal.pone.0291074 (PMC10530016; doi:10.1371/journal.pone.0291074)
Supplement: S12 Table — (DOCX) [file pone.0291074.s013.docx]

**Table S12. Errors (2σ) on determination of past soil CO_2_ from Jurta paleosols**

| Location | pCO_2_  (ppm) | ± ppm | Cation loss  (± ppm) | Age (±ppm) | Henry’s Law constant (± ppm) | Mean annual precipitation (± ppm) | Diffusion constant (± ppm) | Diffusion constant ratio soil/air (± ppm) |
| --- | --- | --- | --- | --- | --- | --- | --- | --- |
| Strelley Pool | 3170 | 446 | 446 | 1.05 | 8.34x10^-9^ | 6.38x10^-6^ | 1.71x10^-7^ | 5.78x10^-13^ |
| Trendall Rdige | 2011 | 237 | 237 | 2.44 | 1.43x10^-8^ | 7.38x10^-6^ | 4.08x10^-7^ | 9.60x10^-13^ |
| Marble Bar | 2473 | 134 | 134 | 0.95 | 7.20x10^-9^ | 7.10x10^-6^ | 1.51x10^-7^ | 4.01x10^-13^ |

*Note: “A” is duration of soil formation which controls error, and estimate with age is based on thickness of solum from modern pyritic shale weathering rate of (Ma et al., 2010), itemised for these paleosols in Table S12. Ranges for constants and precipitation are in Table S4, and for cation loss from analytical errors of Table S3.*
