# Supplementary material for: Archean (3.3 Ga) paleosols and paleoenvironments of Western Australia
Source: PLoS One. 2023 Sep 27;18(9):e0291074. doi: 10.1371/journal.pone.0291074 (PMC10530016; doi:10.1371/journal.pone.0291074)
Supplement: S13 Table — (DOCX) [file pone.0291074.s014.docx]

**Table S13. Errors (2σ) on determination of past soil O_2_ from Jurta paleosols**

| Location | pO_2_  method herein  (ppm) | ± ppm | Cation loss  (± ppm) | Age (± ppm) | Henry’s Law constant (± ppm) | Mean annual precipit-ation (± ppm) | Diffusion constant (± ppm) | Diffusion constant ratio soil/air (± ppm) | Holland’s R (O_2_/CO_2_ demand | pO_2_ (ppm)  method of Grandstaff et al., (1986) |
| --- | --- | --- | --- | --- | --- | --- | --- | --- | --- | --- |
| Strelley Pool | 1788 | 4006 | 4006 | 14.78 | 1.16x10^-9^ | 4.21x10^-5^ | 3.32x10^-7^ | 1.58x10^-8^ | 0.034 | 6.5±6.2 |
| Trendall Ridge | 1124 | 5085 | 5085 | 4.42 | 1.93x10^-9^ | 7.03x10^-5^ | 7.70x10^-7^ | 2.55x10^-8^ | 0.037 | 4.1±3.9 |
| Marble Bar | 2181 | 3018 | 3018 | 8.22 | 6.36x10^-9^ | 2.31x10^-5^ | 1.84x10^-7^ | 8.22x10^-10^ | 0.017 | 5.1±4.8 |

*Note: Estimate with age is based on thickness of solum from modern chronofunction of Table S4, itemised for these paleosols in Table S12. Ranges for constants precipitation are in Table S4, and for cation loss from errors of Table S3. Error for method of Grandstaff et al. (1986) is bounds of equilibrium and diffusion limits, so is a range rather than formal error.*
